# Partial Sleep Restriction Activates Immune Response-Related Gene Expression Pathways: Experimental and Epidemiological Studies in Humans

**DOI:** 10.1371/journal.pone.0077184

**Published:** 2013-10-23

**Authors:** Vilma Aho, Hanna M. Ollila, Ville Rantanen, Erkki Kronholm, Ida Surakka, Wessel M. A. van Leeuwen, Maili Lehto, Sampsa Matikainen, Samuli Ripatti, Mikko Härmä, Mikael Sallinen, Veikko Salomaa, Matti Jauhiainen, Harri Alenius, Tiina Paunio, Tarja Porkka-Heiskanen

**Affiliations:** 1 Department of Physiology, Institute of Biomedicine, University of Helsinki, Helsinki, Finland; 2 Department of Molecular Medicine, National Institute for Health and Welfare, Helsinki, Finland; 3 FIMM, Finnish Institute of Molecular Medicine, Helsinki, Finland; 4 Department of Psychiatry, HUCH, Helsinki, Finland; 5 Research Programs Unit, Genome-Scale Biology & Institute of Biomedicine, University of Helsinki, Helsinki, Finland; 6 Department of Chronic Disease Prevention, National Institute for Health and Welfare, Helsinki, Finland; 7 Centre of Expertise for Human Factors at Work, Finnish Institute of Occupational Health, Helsinki, Finland; 8 Stress Research Institute, Stockholm University, Stockholm, Sweden; 9 Unit of Systems Toxicology, Centre of Expertise for Health and Work Ability, Finnish Institute of Occupational Health, Helsinki, Finland; 10 Department of Medical Epidemiology & Biostatistics, Karolinska Institute, Stockholm, Sweden; 11 Department of Human Genetics, Wellcome Trust Sanger Institute, Wellcome Trust Genome Campus, Hinxton, United Kingdom; 12 Agora Center, University of Jyväskylä, Jyväskylä, Finland; Université de Montréal, Canada

## Abstract

Epidemiological studies have shown that short or insufficient sleep is associated with increased risk for metabolic diseases and mortality. To elucidate mechanisms behind this connection, we aimed to identify genes and pathways affected by experimentally induced, partial sleep restriction and to verify their connection to insufficient sleep at population level. The experimental design simulated sleep restriction during a working week: sleep of healthy men (N = 9) was restricted to 4 h/night for five nights. The control subjects (N = 4) spent 8 h/night in bed. Leukocyte RNA expression was analyzed at baseline, after sleep restriction, and after recovery using whole genome microarrays complemented with pathway and transcription factor analysis. Expression levels of the ten most up-regulated and ten most down-regulated transcripts were correlated with subjective assessment of insufficient sleep in a population cohort (N = 472). Experimental sleep restriction altered the expression of 117 genes. Eight of the 25 most up-regulated transcripts were related to immune function. Accordingly, fifteen of the 25 most up-regulated Gene Ontology pathways were also related to immune function, including those for B cell activation, interleukin 8 production, and NF-κB signaling (*P*<0.005). Of the ten most up-regulated genes, expression of *STX16* correlated negatively with self-reported insufficient sleep in a population sample, while three other genes showed tendency for positive correlation. Of the ten most down-regulated genes, *TBX21* and *LGR6* correlated negatively and *TGFBR3* positively with insufficient sleep. Partial sleep restriction affects the regulation of signaling pathways related to the immune system. Some of these changes appear to be long-lasting and may at least partly explain how prolonged sleep restriction can contribute to inflammation-associated pathological states, such as cardiometabolic diseases.

## Introduction

In addition to compromised brain function, restriction of sleep has many adverse effects on human physiology and health. Epidemiologic studies have shown an association between self-reported sleep duration and cardiometabolic diseases: sleep duration that deviates from 7–8 h per night is associated with several cardiovascular risk factors, including elevated blood pressure, increased heart rate [1], coronary heart disease [2], [3], obesity [4], and type II diabetes [5], [6]. Both overall mortality and mortality of cardiovascular diseases are increased in individuals who sleep less than 7 hours [7]–[10].

Experimental sleep restriction (SR) studies have provided data that give some insight into the potential mechanism that may explain the increase in cardiometabolic diseases. Increased blood pressure and heart rate during and after sleep restriction has been a frequent finding in studies where sleep has been totally or partially restricted [3], [11], [12]. Possible metabolic consequences of sleep restriction include the development of insulin resistance, a state that precedes type II diabetes [13], increase of serum ghrelin levels and decrease [13], [14] or increase [15] of leptin levels. These changes may contribute to the increased food intake during SR and predispose to development of obesity [13].

Experimental sleep restriction studies conducted in humans and using animal models consistently show activation of immune defense during sleep restriction. Increased levels of pro-inflammatory cytokines [16]–[18] and C-reactive protein (CRP) [19], [20] as well as activation of nuclear factor kappa B (NF-κB) [21], [22] have been reported. Prolonged low level activation of these inflammatory markers is also associated with several chronic diseases, including cardiovascular diseases and type II diabetes [23]. Thus there is compelling evidence on the connection between SR, activation of immune function-related molecular pathways and cardiometabolic diseases.

We have previously reported that partial SR increased serum levels of CRP, changed the numbers of blood leukocytes, and activated the peripheral blood mononuclear cells [19]. The activation was evidenced as increased gene expression and protein levels of selected cytokines, interleukins (ILs) 1β, 6, and 17, as response to *in vitro* immunological challenge. These extensive changes in the immune responses prompted us to further characterization of the patterns using genome-wide gene expression analysis.

One important question that has remained largely unexplored concerns the relationship between findings produced in short-term experimental studies and the real life exposure to sleep restriction, which mostly can be characterized as partial and long-term. The epidemiologic data is based on the latter condition, and it is important to build bridges between the experimentally created data and the data collected in epidemiologic research from real life conditions.

In the present study we used two strategies to decrease this gap: 1) the experimental part of the study was planned to mimic real life conditions, and 2) we collected biological data, including gene expression data, from an epidemiologic cohort. We believe that combining these data sets will increase our understanding also on the relationships between experimental and real life conditions.

In the experimental part, sleep of healthy volunteers was restricted to 4 hours per day during five days, followed by two nights of recovery sleep. We have earlier reported changes in glucose metabolism [15], and cytokines, white blood cell subpopulations, and C-reactive protein [19] from this experiment). Gene expression was assessed using whole genome microarrays at baseline, after the SR period, and after recovery. These conditions were compared within the subjects as well as with the control group who spent the same time in the laboratory but spent eight hours per night in bed. In the population study, the most significantly affected genes, identified in the experimental study, were correlated with self-reported insufficient sleep as an indicator of sleep restriction in a Finnish population cohort of 472 individuals.

To the best of our knowledge, this is the first study to address changes in gene expression at whole genomic level in response to partial, cumulative sleep loss in humans, and insufficient sleep at population level.

## Results

Changes in gene expression in the experimental SR study were assessed in peripheral blood mononuclear cells (PBMC) with genome-wide microarrays. The microarray data were analyzed at two levels: at single gene level and at gene network level.

The single gene analysis consisted of identifying transcripts that were differently expressed after SR. We chose to perform a conservative, stepwise analysis aiming to retain transcripts with significant effect but modest *P* value in the analysis.

The network analysis aimed to find biological gene networks that were significantly changed after SR.

### Analysis of the single transcripts

**Figure 1:**
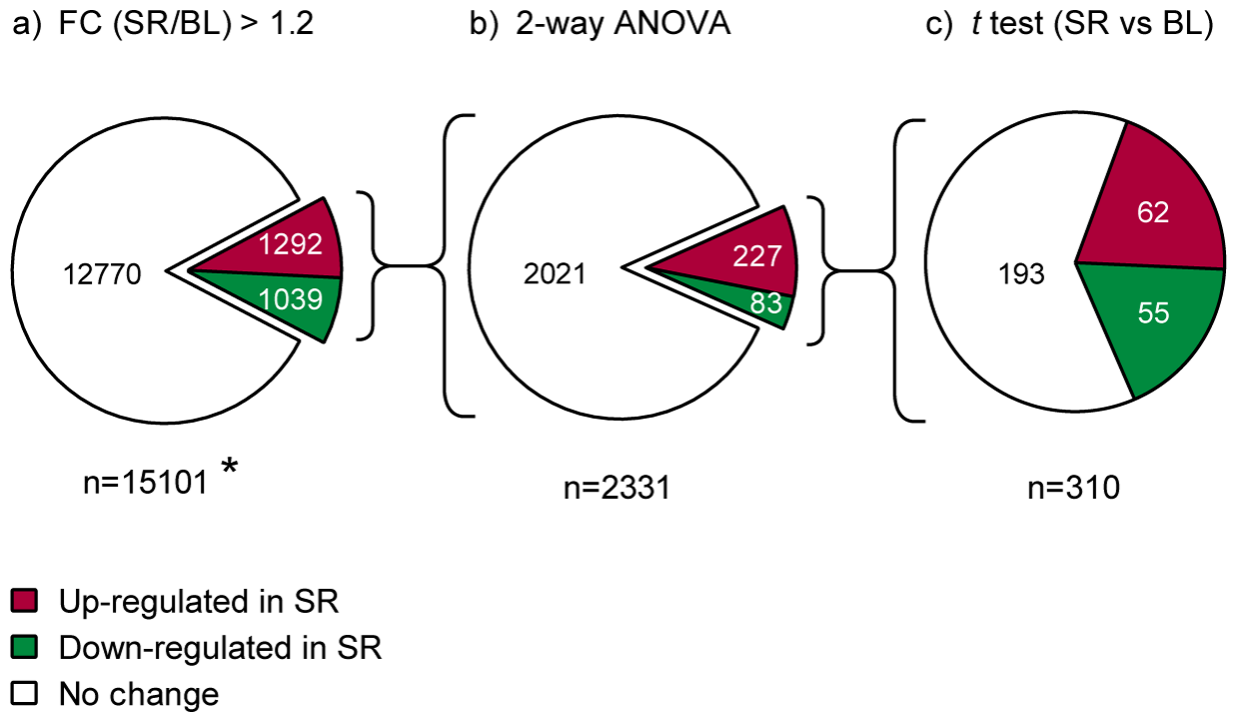
Analysis strategy. The flow of statistical analysis and the amount of entities (genes/transcripts) after each step are illustrated. a) The 15101 entities that passed the filtering by flags and reannotation included 1292 up-regulated (red) and 1039 down-regulated (green) transcripts with at least 1.2-fold change from baseline (BL) to sleep restriction (SR) in sleep-restricted subjects ( = cases). * The pathway analysis was run for these genes. b) The 2331 entities were analyzed with 2-way ANOVA using the case/control status and the three timepoints as analysis axes. Changes with ANOVA interaction *P* value <0.05 were observed in 227 up-regulated and 83 down-regulated transcripts. c) Altogether 310 entities were further analyzed with 1-way repeated measures ANOVA considering the three timepoints. The 43 entities showing changes also in the control group were excluded from the analysis. The 133 genes with 1-way ANOVA *P* value <0.05 for the cases but not for the controls were then analyzed using a *t* test between the timepoints BL and SR. 62 genes were up-regulated and 55 down-regulated (P<0.05).

The final probe filtering and data analysis was performed stepwise in three phases (see **Figure 1**).

Phase 1 (**Figure 1a**). The expression levels of altogether 2331 transcripts (1292 up-regulated and 1039 down-regulated) were changed with at least 1.2-fold after 5 nights of partial SR compared to BL (**Figure 1a**).

**Figure 2:**
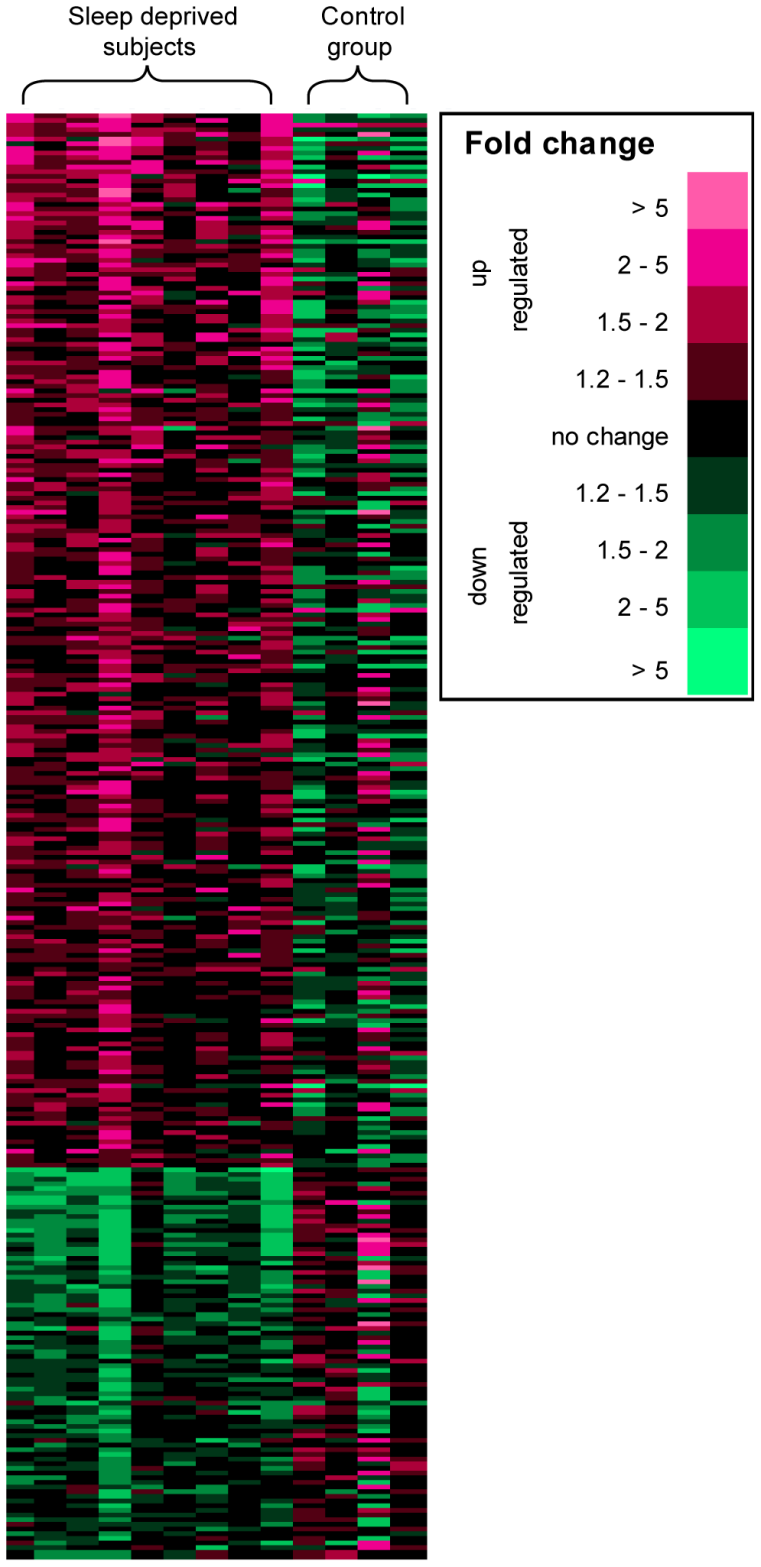
Expression changes after partial sleep restriction. The 310 entities (genes/transcripts) with interaction *P* value (*P*<0.05) in 2-way ANOVA, sorted by average fold change from baseline (BL) to sleep restriction (SR) (with the up-regulated (red) on top, followed by the down-regulated (green). Each lane represents one individual (sleep deprived subjects, N = 9; controls, N = 4), and colour codes represent the fold change from BL to SR (BL  = 1).

Phase 2 (**Figure 1b**). 2-way ANOVA. In order to evaluate the statistical significance of the differentially expressed genes, in the first phase 2-way repeated measures ANOVA was adopted (R/Bioconductor open software packages; http://www.r-project.org) covering the three timepoints (BL, SR, REC) and the case/control group, as well as their interaction. A conservative cut-off of *P*<0.05 (for the interaction) was used to filter out most of the transcripts, leaving 310 for further analyses. Of these, 227 were up-regulated and 83 down-regulated. The fold change differences are presented in **Figure 2** and the genes are listed in **Tables S1** and **S2**.

Phase 3 (**Figure 1c**). 1-way ANOVA and *t* tests. To eliminate the changes occurring also in the control subjects, the analysis from phase 2 was continued with 1-way repeated measures ANOVA of the timepoint axis, analyzing the experimental group and the control group separately. The individual timepoints were compared using paired *t* tests, comparing the SR and REC timepoints to BL. Between BL and SR, the expression of 117 transcripts was changed in the experimental group, but not in the control group. 62 transcripts were up-regulated and 55 down-regulated. The top 25 up- and down-regulated transcripts are listed in order of fold change from BL to SR in **Tables 1** and **2**, respectively. Most of the expression changes returned back to BL levels during two nights of 6 recovery, but 14 genes remained up-regulated and 6 down-regulated in REC (paired *t* test, pointwise *P*<0.05).

**Table 1:** Up-regulated genes after cumulative sleep restriction.

		SR to BL	
Affymetrix Probe	Gene symbol	*P* value	Fold change
221060_s_at	**TLR4**	0.0218	1.91
210148_at	**HIPK3**	0.0016	1.91
214590_s_at	**UBE2D1**	0.0047	1.71
216901_s_at	**IKZF1**	0.0031	1.71
201235_s_at	**BTG2**	0.0029	1.65
210773_s_at	**FPR2**	0.0069	1.63
221638_s_at	**STX16**	0.0030	1.63
221239_s_at	**FCRL2**	0.0033	1.58
203923_s_at	**CYBB**	0.0227	1.53
215159_s_at	**NADK**	0.0056	1.50
202874_s_at	**ATP6V1C1**	0.0008	1.46
227697_at	**SOCS3**	0.0016	1.46
224917_at	**MIRN21**	0.0149	1.45
1552787_at	**HELB**	0.0030	1.44
224760_at	**SP1**	0.0015	1.43
210772_at	**FPR2**	0.0074	1.39
201669_s_at	**MARCKS**	0.0248	1.38
241734_at	**SRFBP1**	0.0029	1.37
210872_x_at	**GAS7**	0.0123	1.34
209306_s_at	**SWAP70**	0.0003	1.34
208650_s_at	**CD24**	0.0027	1.33
217208_s_at	**DLG1**	0.0235	1.33
206359_at	**SOCS3**	0.0043	1.32
201971_s_at	**ATP6V1A**	0.0029	1.32
231955_s_at	**HIBADH**	0.0369	1.31

The expression changes of the 25 most up-regulated genes/transcripts after experimental sleep restriction period (SR) compared to baseline values (BL). Pointwise *P* values and fold changes (SR expression/BL expression) between these two timepoints are shown for each transcript.

**Table 2:** Down-regulated genes after experimental sleep restriction.

		SR to BL	
Affymetrix Probe	Gene symbol	*P* value	Fold change
211687_x_at	**KIR3DL1**	0.0036	0.46
210164_at	**GZMB**	0.0017	0.54
212843_at	**NCAM1**	0.0004	0.57
228774_at	**CEP78**	0.0020	0.59
210321_at	**GZMH**	0.0019	0.59
220646_s_at	**KLRF1**	0.0055	0.60
227819_at	**LGR6**	0.0041	0.60
228063_s_at	**NAP1L5**	0.0050	0.61
37145_at	**GNLY**	0.0015	0.61
205495_s_at	**GNLY**	0.0019	0.62
207072_at	**IL18RAP**	0.0060	0.63
226625_at	**TGFBR3**	0.0040	0.64
226858_at	**CSNK1E**	0.0179	0.65
210140_at	**CST7**	0.0029	0.65
220684_at	**TBX21**	0.0022	0.66
213915_at	**NKG7**	0.0048	0.66
206267_s_at	**MATK**	0.0011	0.67
202146_at	**IFRD1**	0.0206	0.68
205291_at	**IL2RB**	0.0093	0.68
209993_at	**ABCB1**	0.0048	0.69
1553736_at	**CCDC131**	0.0009	0.70
214450_at	**CTSW**	0.0041	0.70
235232_at	**GMEB1**	0.0040	0.71
214470_at	**KLRB1**	0.0046	0.72
224315_at	**DDX20**	0.0318	0.72

The expression changes of the 25 most down-regulated genes/transcripts after experimental sleep restriction period (SR) compared to baseline values (BL). Pointwise *P* values and fold changes (SR expression / BL expression) between these two timepoints are shown for each transcript.

Of the top 25 most up-regulated transcripts, at least 8 (*TLR4, IKZF1, FPR2, FCRL2, CYBB, SOCS3, SWAP70, DLG1*) have been annotated to immune-related GO pathways. There were also several stress and/or apoptosis-related transcripts (*HIPK3, BTG2, SRFBP1*) among the most up-regulated transcripts.

Of the top 25 most down-regulated genes, 19 were immune-related, mostly related to natural killer cell (NK cell) function (such as *KIR3DL1, NCAM1, GZMB, GZMH, KLRF1, NKG7, KLRB1, DDX20, GNLY*). As the number of NK cells was reduced during SR, the reduction in the expression level of these genes could be a consequence of this reduction.

The expression levels of six of the genes related to immune functions or stress/apoptosis (*BTG2, FCRL2, HIPK3, IKZF1, STX16,* and *TGFBR3*) were validated with quantitative PCR (qPCR) analysis. Four of these (*BTG2, FCRL2, IKZF1, and STX16*) were up-regulated and one (*TGFBR3*) down-regulated after SR (*P*<0.05), confirming the results obtained with the microarray analysis (**Table S3**).

### Transcription factor binding site analysis

Enrichment of binding sites for specific transcription factors among the up- or down-regulated genes was analyzed using the over-representation analysis tool oPOSSUM. Human single site analysis was applied as described in [24].

Binding sequences for eight vertebrate transcription factors (Prrx2, IRF2, Nobox, NFYA, STAT1, Pdx1, SRY, and Pax5) were significantly over-represented in the up-regulated transcripts (one-tailed Fisher exact probability test *P*<0.05). Of the genes coding for these transcription factors, interferon regulatory factor 2 (*IRF2*; pointwise *P* = 0.020, and fold change (FC)  = 1.50) and signal transducer and activator of transcription 1 (*STAT1*; pointwise *P* = 0.027, FC = 1.38) showed higher expression after SR (compared to BL with *t* test) in the microarray analysis.

Among the down-regulated transcripts, binding sequences for five transcription factors (PBX1, Fos, RELA, GABPA, MYC-MAX, and Nobox) were enriched (*P*<0.05). None of the mRNAs coding for these were decreased after SR in the microarray analysis (*Fos* expression was up-regulated; pointwise *P* = 0.015, FC = 2.80).

### Analysis of biological networks

In order to characterize which biological pathways were enriched, a nonparametric in house developed CIGA pathway analysis program was used. This program uses Gene Ontology (GO) annotations that group the single genes in biologically meaningful pathways [25]. The GO pathways were assessed among all transcripts that had passed the quality control (N = 15 101).

*Up-regulated pathways* included highly significant enrichment of inflammation and immunity related GOs (*P*<0.001, *P*<0.05 after permutation) (**Tables 3** and **S4**). Two major functional groups were identified: the activation of leukocytes and adaptive immunity, and the activation of cytokine pathways. The most significant pathways were B cell activation and interleukin 8 production (*P*<10^−5^, *P*<0.001 after permutation; the following *P* values given are those obtained after permutation).

**Table 3:** Gene Ontology pathways up-regulated after sleep restriction.

Pathway	*P* value	Total genes	Gene rank	Top genes	Contributing genes
B cell activation	0.001	90	1868	43	AC, COPEB, LIG4, CD24L4, SKAP2, MS4A1, BCL6, MS4A1, MS4A1, CD79A
interleukin-8 production	0.001	9	1282	8	BPI, TLR4, TLR8, BCL10, TLR7
lipopolysaccharide binding	0.001	9	1282	8	TLR2, PTAFR, BPI, TLR4, PTAFR
xenobiotic metabolic process	0.001	8	352	5	DEFA4, KYNU, S100A12
coagulation	0.001	75	821	22	C3AR1, NELL2, PLSCR1, ANXA5, ENTPD1, NID1, PTAFR, PLXDC2, RAB27A, CD36
leukocyte activation	0.001	208	1499	68	AC, COPEB, CTLA4, HLX, LIG4, CD24L4, SKAP2, MS4A1, BCL6, CD86
cell activation	0.002	217	1499	70	AC, COPEB, CTLA4, PLSCR1, HLX, LIG4, CD24L4, SKAP2, MS4A1, BCL6
adaptive immune resp. based on somatic recomb. of immune receptors built from IG superfamily domains	0.002	83	2853	51	AC, HLX, LIG4, CD24L4, BCL6, CD86, RAB27A, CR1, MYD88, LY9
phospholipid binding	0.003	91	1083	29	DGKA,ANXA5, OSBPL1A, SNX3, PTAFR, DGKA, SGK1, DAPP, ANXA2, EPB41
lymphocyte activation	0.002	184	1499	61	AC, COPEB, CTLA4, HLX, LIG4, CD24L4, SKAP2, MS4A1, BCL6, CD86
positive regulation of interleukin-8 biosynthetic process	0.001	8	1282	7	TLR4, TLR8, BCL10, TLR7
interleukin-8 biosynthetic process	0.001	8	1282	7	TLR4, TLR8, BCL10, TLR7
regulation of interleukin-8 biosynthetic process	0.001	8	1282	7	TLR4, TLR8, BCL10, TLR7
oxygen transport	0.001	10	1329	8	HBG1, HBA2
hemoglobin complex	0.001	10	1329	8	HBG1, HBA2
adaptive immune response	0.001	88	2853	53	AC, HLX, LIG4, CD24L4, BCL6, CD86, IL6ST, RAB27A, CR1, MYD88
leukocyte differentiation	0.002	110	2624	60	COPEB, CTLA4, HLX LIG4, CD24L4, BCL6, CD86, CD79A, CTLA4
response to xenobiotic stimulus	0.003	10	352	5	DEFA4, KYNU, DEFA1, S100A12
Arp2/3 protein complex	0.001	15	3855	15	ACTR2, ACTR3, ARPC5, ARPC1B, TTLL3
lymphocyte differentiation	0.005	93	1482	35	COPEB, CTLA4, HLX LIG4, CD24L4, BCL6, CD86, CD79A, CTLA4, ZEB1
I-kappaB kinase/NF-kappaB cascade	0.003	107	1499	39	AZI2, TLR2, UBE2N, LY96, CFLAR, TIFA, RP11-119B16.1, MYD88, CASP1, CD40
positive regulation of cytokine biosynthetic process	0.003	26	2593	19	CD86, MYD88, SYK, TLR1, TLR4, TLR8, BCL10, TLR7, STAT5B, CD86
gas transport	0.001	11	1329	8	HBG1, HBA2, RP3-402L9.2,CA2
blood coagulation	0.006	65	588	15	C3AR1, NELL2, PLSCR1, ANXA5, ENTPD1, NID1, PTAFR, PLXDC2, RAB27A, CD36
response to fungus	0.001	17	1282	10	DEFA4, TLR2, DEFA1, S100A12, MYD88, TLR4, BCL10, CLEC7A, COTL1, PTX3

The top 25 Gene Ontology pathways (biological processes) that were significantly enriched (*P*<0.01 after permutation) among the transcripts up-regulated after sleep restriction. *Total genes* represents the number of genes that are annotated to the pathway. *Top genes* represents the number of genes that were found in the study setting and contributed to the significance of the pathway.

As a feature of the tree-like structure of the GO classifications, many of the pathways overlap. Of the 25 most significant pathways, genes comprising eight pathways related to leukocyte activation and adaptive immune response (B cell activation, leukocyte activation, cell activation, lymphocyte activation, adaptive immune response, adaptive immune response based on somatic recombination of immune receptors built from immunoglobulin superfamily domains, leukocyte differentiation, and lymphocyte differentiation) overlapped considerably (**Table 3**). The ten most frequent genes from these lymphocyte activation-related pathways (8 pathways altogether) were: *CD24L4, COPEB (KLF6), CTLA4, AC, MS4A1, LIG4, CD86, SKAP2, HLX,* and *BCL6*.

In the activation of cytokine pathways group the overlapping genes comprised of six pathways (IL-8 biosynthesis, positive regulation of IL-8 biosynthesis, IL-8 production, lipopolysaccharide binding (all *P*<0.001), and positive regulation of cytokine biosynthesis (*P*<0.005)). The ten most frequent transcripts from the cytokine biosynthesis pathways (altogether 6 pathways) were: *TLR4, TLR8, BCL10, BPI, PTAFR, CD86, TLR2, MYD88, SYK,* and *TLR1*.

Also xenobiotic process (*P*<0.001) and response to xenobiotic stimulus (*P*<0.005), I-kB kinase–NF-κB cascade (*P*<0.005), and T helper 2 cell differentiation (*P*<0.01) were among the significantly enriched pathways.

In addition to the immunological pathways, we observed significant enrichment of GOs related to blood coagulation (*P*<0.001), oxygen transport (oxygen transport, gas transport (*P*<0.001)), and hemoglobin catabolism (hemoglobin complex (*P*<0.001), heme catabolic process (*P*<0.001), pigment catabolic process (*P*<0.005)). Also phospholipid binding (*P*<0.005) and Arp2/3 complex (*P*<0.001) were enriched among the up-regulated transcripts.

*Down-regulated pathways* included an enrichment of a biologically different set of GOs, listed in **Table S5**. These included pathways participating in lipid transport; intracellular lipid transport, cholesterol efflux, sequestering of lipid, and sterol transporter activity (*P*<0.001) being the top-ranked. Altogether more than half of the first 25 top-ranked pathways were related to lipid metabolism or transport. Only one directly immune-related pathway was among this group: the MHC class I receptor activity pathway.

### Interferon γ in stimulated leukocytes

Stimulation of PBMC with phytohaemagglutinin (PHA) resulted in lower level of interferon-γ (IFN-γ) protein in samples taken after SR (mean ± SD 5.34±2.39 µg/l) compared to the cells stimulated after BL conditions (4.41±2.27 µg/ml) (*P* = 0.049).

### RNA expression in the population sample

The National FINRISK Study is a cross-sectional study performed every fifth year since 1972. Its aim is to evaluate the cardiovascular risk factors in Finland using questionnaires, laboratory measurements, and information on general health of the participants [26]. Lifestyle and Genetic determinants of Obesity and Metabolic Syndrome (DILGOM) study was performed as an extension of the FINRISK 2007 study comprising also whole genome RNA expression data from altogether 518 individuals (age 25–74 years). One question in the questionnaire was particularly aimed at estimating the grade of sleep debt: “*Do you, in your opinion, sleep enough?*” with answering options: 1) “*Yes, almost always”* (N = 168), 2) “*Yes, often”* (N = 218), 3) “*Seldom or almost never”* (N = 86), and 4) “*I cannot say”* (N = 46). Answer 4 was excluded, and answers 1–3 (N = 472) were dichotomized combining 1 and 2 to a phenotype of ‘self-reported sufficient sleep’ and comparing this to 3, ‘self-reported insufficient sleep’. All individuals with genome-wide Illumina RNA expression and sleep insufficiency data (N = 472 individuals from DILGOM) were included in the RNA expression correlation analysis.

The expression of three of the ten most up-regulated genes from the experimental sleep restriction showed a trend for correlation with self-reported insufficient sleep among the DILGOM subjects: *TLR4* (*P* = 0.026, β = +0.27, *P*_corrected_ = 0.26), *HIPK3* (*P* = 0.006, β = +0.90, *P*_corrected_ = 0.059), and *FCRL2* (*P* = 0.018, β = +1.02, *P*_corrected_ = 0.18), but it was not statistically significant after correction with the number of genes analyzed in the population sample (N = 10).

The expression of *STX16* was significantly lower among the subjects with insufficient sleep (*P* = 0.0002, β = −1.31, *P*_corrected_ = 0.002), while in the experimental sleep restriction study it was among the 10 most up-regulated genes.

Three of the ten most down-regulated genes from the experimental sleep restriction group correlated with self-reported insufficient sleep in the DILGOM sample. The expressions of *TBX21* (*P* = 1.8*10^7^, β = −0.78, *P*_corrected_ = 1.8*10^6^) and *LGR6* (*P* = 0.0007, β = −0.79, *P*_corrected_ = 0.007) were significantly lower among the subjects reporting insufficient sleep, while *TGFBR3* expression was higher in the group with insufficient sleep (*P* = 0.0001, β = +0.41, *P*_corrected_ = 0.001). Also *KIR3DL1* (*P* = 0.028, β = +0.26, *P*_corrected_ = 0.28) and *GZMB* (*P* = 0.009, β = +0.17, *P*_corrected_ = 0.092) showed a trend for higher expression with insufficient sleep, but these findings were not statistically significant after correction for multiple testing.

### C-reactive protein levels in the population sample

To examine whether the activation of the acute phase response, seen in the experimental study as elevated CRP (19), was also present in the population sample, we studied altogether 6803 individuals from the FINRISK 2007 that had answered the question about sleep sufficiency (above) and had laboratory measurements of serum CRP levels.

There was a significant association with CRP levels and self-reported insufficient sleep among males. Males reporting insufficient sleep showed moderately, but statistically significantly, higher CRP levels (mean ± SD 2.61±7.02 mg/l), than those who reported that they were well rested (1.92±4.13 mg/l) (*P* = 0.0017). No significant difference was found in females.

## Discussion

The main finding of the study was that experimental sleep restriction for one working week extensively activates the immune system, up-regulating many immune response-related gene pathways and individual gene transcripts. Importantly, some of the same genes that increased/decreased their expression in experimental SR were affected also at population level in individuals who reported insufficient sleep.

### Lymphocyte activation

#### B cells

**Figure 3:**
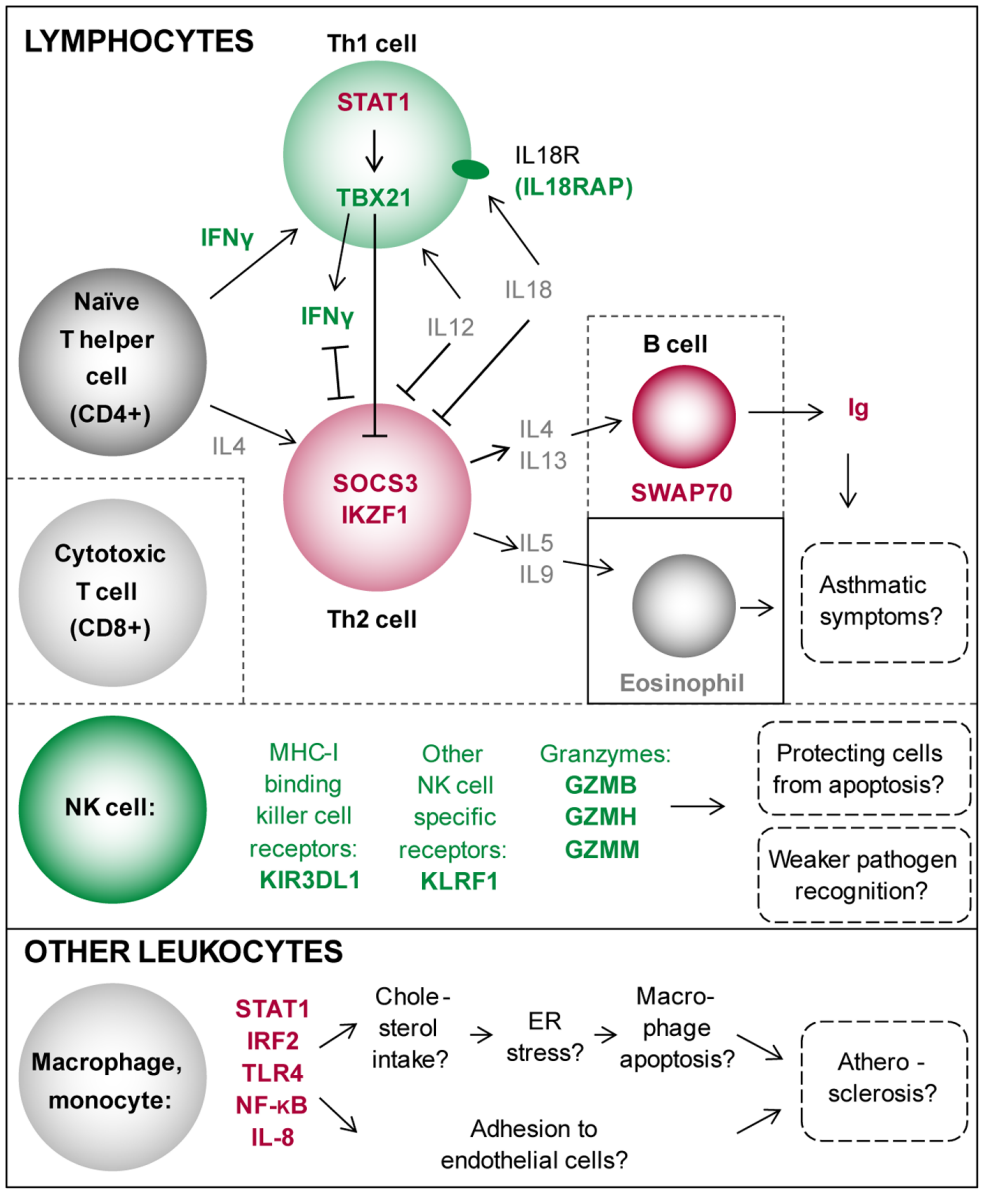
Differentiation and activation of lymphocyte and other leukocyte populations in cumulative sleep restriction (SR). Arrows (↑) illustrate activating and blunt ended lines (T) inhibiting effects. Red  =  increased after SR, green  =  decreased after SR, grey  =  no change observed. IFNγ  =  interferon γ, IL  =  interleukin, STAT1, TBX21 and SOCS3  =  transcription factors, Ig  =  immunoglobulins. (The factors involved in T helper (Th) cell differentiation modified after [30]). We propose that cumulative SR via the activation of B cell-mediated humoral immunity by Th2 cells may lead to increased risk for development or exacerbation of asthmatic symptoms. We also suggest that SR may participate in the development of atherosclerosis through increased cholesterol intake into macrophages. On the other hand, the reduction in the NK cell type immune response may be a mode to protect the self from destruction by apoptosis and/or might contribute to the attenuated immune response towards pathogens.

In the pathway analysis, the enrichment of genes involved in B lymphocyte activation (pathway “B cell activation”) was most significant, indicating the activation of the humoral immune system. Supporting this, we found also the pathway of “adaptive immune response based on somatic recombination of immune receptors built from immunoglobulin superfamily domains” among the top ranked pathways. Of the individual up-regulated transcripts, *SWAP70* is specifically expressed in B cells, promoting B cell activation through immunoglobulin (Ig) G binding as well as migration and adhesion of B cells [27].These findings are in line with our previous observation showing that the amount of B cells was increased in cumulative SR [19] and other reports showing that parameters of humoral immunity, including serum immunoglobulins (IgG, IgA, IgM), were elevated in sleep deprivation [28]. The current results suggest that these sleep restriction-induced changes in humoral immunity are regulated also at the level of gene expression (**Figure 3**).

#### T cells

We observed the activation of T cells at transcriptional level after SR. We found signs of activation of the T helper cell 2 (Th2) type immune response pathways, which activate B cell-mediated immunity. Accordingly, transcription factors *SOCS3* and *IKZF1*, which drive Th2-development, were among the most up-regulated transcripts after SR. However, the number of T-cells was not changed (19).

T helper cell 1 (Th1) and Th2 lineages are reciprocally inhibited. Accordingly, we found down-regulation of the T-box transcription factor *TBX21* (*T-bet*), the key regulator of the Th1-differentation. TBX21 induces the production of the Th1-driving cytokine IFN-γ [29]. When we stimulated the leukocytes with T cell-stimulating PHA and measured cytokine production, the production of IFN-γ was lower in the samples taken after SR suggesting that SR may have a direct functional significance on IFN-γ via TBX21-mediated gene expression. Importantly, the expression of *TBX21* was down-regulated also in individuals who reported insufficient sleep, implying down-regulation of the Th1 type immunity also at the population level (for more detailed discussion, see below).

These findings suggest that cumulative SR may drive the immune response towards Th2 cell type and further to B cell activation and humoral immune response by inhibiting the Th1 activation line and by favoring the Th2 activation line (**Figure 3**).

### Innate immunity-related findings

#### NK cells

NK cells form a part of the innate immune system and are capable of recognizing infected or damaged cells. We and others have previously reported a decrease in the number of NK cells after SR [19], [31]. In the present study, the pathway “MHC class I receptor activity” was down-regulated after SR. This was the only immune function related pathway that was down-regulated. The MHC-I complex interacts with cytotoxic T cells and NK cells, presenting mainly short peptides that originate within the cell. The pathway consists mostly of genes coding for killer cell immunoglobulin-like receptors. When the MHC-I expression is down-regulated on target cells, the NK cells may activate and destroy them (reviewed in [32]). The process is mediated by secretion of cytotoxic proteases, such as granzymes, which activate the caspase cascade in the target cells, eventually leading to apoptosis [33]. We observed a down-regulation of genes coding for granzymes B, H, and M (*GZMB, GZMH, GZMM*) after SR, which may indicate decreased NK cell function. This may be a way of protecting the cells from apoptosis, and/or contribute to the weaker response in pathogen recognition and attenuated immunity towards infections seen in SR [34] (**Figure 3**). As the genes contributing significantly to the MHC-I pathway are expressed in the NK cells we cannot rule out the possibility that the reduced number of NK cells explains the down-regulation of this pathway.

More than half of the 25 most down-regulated pathways were related to cholesterol/lipid metabolism and transport. In addition, several kinase pathways and a circadian rhythm pathway were down-regulated. The changes induced by partial sleep restriction in these functions remain a topic for further investigation.

#### IL-8, TLR4, NF-κB, and STAT1

The second most significantly enriched pathway among the up-regulated genes was “interleukin 8 production”. IL-8 acts as a mediator of acute inflammatory response by recruiting monocytes and neutrophils (reviewed in [35]). The major sources of IL-8 are monocytes and macrophages, challenged by bacteria, viruses, or pro-inflammatory cytokines (reviewed in [36]). Co-operative activation of transcription factors NF-κB and AP-1 precede the IL-8 gene transcription [36].The IL-8 production pathway up-regulation comprised mainly of members of the family toll-like receptors (TLRs 4, 7, and 8). TLRs recognize conserved pathogen patterns, such as lipopolysaccharide (LPS), but also endogenous ligands. Activation of these TLRs leads to the activation of the NF-κB signaling cascade [37] leading to induction of several pro-inflammatory cytokines, such as TNF-α and type I interferons [38]. NF-κB activation has been observed in many pathological states such as obesity and atherosclerosis [39] but also in SR [21], [22]. In the present study, TLR4 mRNA expression was increased after SR, implying the activation of the early defense of innate immunity pathway(s). Also several other TLRs showed a trend for up-regulation after SR (**Table S6**). Accordingly, at the population level, the expression of TLR4 showed a trend for a positive association with insufficient sleep.

At pathway level, NF-κB signaling was up-regulated after experimental SR. Binding sites for the transcription factors STAT1 and IRF2 were concentrated among the up-regulated transcripts after SR. One possible mechanism leading to NF- κB activation is trough IRF2 mediated signaling, as the binding sequences for IRF2 were enriched among the promoter regions of the up-regulated genes. IRF2 regulates NF-κB activity by modulating its cellular location [40]. Similarly, STAT1 has been suggested to act as a point of convergence for the cross-talk between the pro-atherogenic TLR4, IFN-γ, and IL-6 activated pathways in immune as well as vascular cells, amplifying pro-inflammatory signals, increasing endothelial cell adhesion, and possibly promoting the development of atherosclerosis [41]. Thus, the present study offers further evidence of the activation of the NF-κB pathway during sleep restriction through IRF2 and TLR4 mediated signaling. These changes may be relevant also at population level.

Another function of STAT1, also possibly contributing to the development or exacerbation of atherosclerosis, occurs in the macrophages. STAT1 has been shown to increase the intake of cholesterol into macrophages [42]. Cholesterol uptake depletes calcium stores in the endoplasmic reticulum (ER) and activates the unfolded protein response (UPR) [43], [44]. ER stress and UPR have been shown earlier to be induced also by sleep restriction in the brain of model organisms [45], [46]. Burden from ER stress together with TLR4 and STAT1 activation has been suggested as the mechanism leading to macrophage apoptosis [47] which, especially in late atherosclerotic lesions, can be detrimental (reviewed in [48]).

Also IRF2 has been reported to participate in the regulation of macrophage apoptosis through a STAT1/3-dependent mechanism [49]. These mechanisms may possibly contribute to the increased development of atherosclerosis in SR (**Figure 3**).

#### CRP

We and others have previously reported that experimental SR increases serum CRP levels [19], [20]. The pathway analysis of the present study showed activation of the IL-8 biosynthesis pathways. IL-8 has been shown to mediate the production of CRP and other acute phase proteins [50]. It has been suggested to act with IL-6 as the main acute phase response mediator in patients with myocardial infarction [51]. Chronic, low level increase in CRP has been associated with cardiovascular diseases [52], short sleep duration [53], [54] and sleep restriction [19], [20]. Accordingly, we found elevated CRP levels in men that reported insufficient sleep in the population sample. Associations between higher CRP have earlier been reported in women [53], [55] and adolescents [54] with short sleep duration, and in men with sleep disturbances [56].

We propose that SR may convey its pro-atherogenic responses through an NF-κB- and TLR4-mediated inflammation reaction, IL-8- and CRP-mediated acute phase response, IL-8-mediated leukocyte adhesion to endothelium, and STAT1-mediated changes in macrophages. On the other hand, Th2-type immune response and activation of B cells may contribute to the development or exacerbation of certain inflammatory disease states, such as asthmatic symptoms (**Figure 3**).

In summary, these results indicate that components of both innate immunity and adaptive immune systems become activated in SR, which may be relevant for general health also at the population level.

### Population study findings

Most studies on physiological changes in sleep restriction have been conducted using continuous waking for 1–3 days. The few comparisons between the continuous waking condition and partial sleep restriction for 4–5 days show that some brain functions in humans [57], as well as the immune system in rats [58] react differently to these challenges. Recently, Möller-Evert *et al*. studied the interaction of circadian phase with moderate cumulative sleep deprivation and acute total sleep loss, and the effects of these on blood transcriptome [59]. They reported that moderate sleep restriction (6 h sleep/night) for one week modified the circadian oscillation of gene expression and the response to acute sleep loss.

Even more differences can be expected if the restriction lasts for months and years, as may be the case with the individuals who report insufficient sleep in the population study. During prolonged exposure to sleep restriction, the original changes may become compensated, and even introduce new harmful factors that threaten health. In spite of possible confounding factors, we were able to identify several genes whose expression levels associated to both experimental sleep restriction and subjectively experienced insufficient sleep. Statistically significant associations, corrected for multiple testing, were observed for the transcripts TBX21 and TGFBR3, which are mediators of the immune system, and LGR6 and STX16 that have been repeatedly associated with cancer progression. Our findings highlight the potential role of these individual transcripts in response to SR, both in experimental and in population level.

In addition, suggestive evidence for association at the population level was found for several genes whose expression was changed after experimental SR. The two genes on the top of the list of the up-regulated individual genes in experimental SR were *TLR4* and *HIPK3*. A trend for increase for both genes was observed also in the population study among subjects that reported insufficient sleep. As discussed above, TLR4 and NF-κB are key regulators of the innate immune system. Interestingly, TLR4 knock-out mice express an attenuated response for sleep deprivation [60]. A recent publication reports that TLR4 mRNA is increased in patients with metabolic syndrome [61], which also is a risk factor for cardiometabolic diseases.

Results from the population-based sample show that the expression of some of the genes that changed their expression during experimental sleep restriction correlated with subjectively experienced insufficient sleep. This finding expands the timespan of sleep restriction beyond the experimental restriction and shows that restricted/insufficient sleep affects the same physiological processes also in natural living conditions. Importantly, these findings suggest that detrimental effects of insufficient sleep may be carried out at the population level by the same molecules that were identified in the experimental SR.

Previous epidemiologic studies, based on questions about sleep duration or insomnia and findings such as an increase in CRP, have suggested a causative relationship between cardiometabolic diseases and insufficient sleep (reviewed in [62]). The increases in CRP levels were identified also in our study, and together with the transcriptional evidence of activation of the immune system give further support to the connection between insufficient sleep and cardiometabolic diseases at population level.

The question of the present epidemiologic study targeted directly the sufficiency/insufficiency of sleep. We believe this question may be more relevant since it avoids the problem of natural short sleepers, who form a confounding factor when using short sleep duration as a marker of insufficient sleep. This issue was discussed in a recent paper by Altman *et al*. [63], prompted by results showing that some cardiovascular outcomes associated differently to sleep duration and sleep insufficiency.

It can be noted that the cohort that was used in the genome-wide expression analysis is small for an epidemiological cohort, and thus the results need to be confirmed in a larger cohort.

Taken together, these findings introduce possible mechanisms through which sleep restriction could, by activating immune responses, predispose to conditions where activation of the immune system plays a role in the pathogenesis of the condition. Such diseases include cardiovascular diseases and type II diabetes, whose development is initiated with prolonged, low level inflammation [23].

### Conclusions

This is the first study to address gene expression changes induced by cumulative sleep restriction in humans at whole genome level in experimental conditions and a population sample. Sleep restriction appears to induce a threat for the body, which leads to extensive activation of both the innate and the acquired immune system. A prolonged condition of insufficient sleep maintains this activation, at least partly, leading to chronic, low-level inflammation – a well-known contributor to many detrimental health conditions, including cardiometabolic diseases.

## Materials and Methods

### Experimental SR study participants and design

The SR experiment has been reported by [19]. In short, fourteen healthy men, aged 19–29 (mean ± SD age 23.1±2.5 years), with a regular sleep-wake schedule and habitual sleep duration of 7–9 h participated in the study. During the experiment, the experimental group (N = 9) spent 8 h in bed for the first two nights (baseline (BL), from 11 PM to 7 AM), followed by 5 nights where they spent only 4 h in bed (sleep restriction (SR), from 3 AM to 7 AM) and, finally, again 2 nights of 8 h in bed (recovery (REC), from 11 PM to 7 AM). The control group (N = 5) spent 8 h in bed (11 PM to 7 AM) throughout the entire experiment. Continuous EEG recordings and a continuously present investigator served to monitor that the participants did not sleep during the periods outside those mentioned above. One control subject deviating from normal sleep pattern was excluded in the quality control phase. Total sleep duration decreased in the experimental group during SR accordingly (mean ± SD sleep/night BL 439±20 min, SR 232±5 min) [19]. There was only a modest (16 min) delay in the circadian rhythm measured by the morning peak timing of saliva cortisol [15], and no overall changes in cortisol levels were observed [19].

Meals were standardized and energy-balanced based on the current national recommendations, provided at fixed times and consumed by all participants throughout the experiment. During waking, the participants could not leave the building but took part in activities simulating a working week (including psychomotor vigilance task, memory and motor tasks etc.). The study design was approved by the ethics committee of the Hospital District of Helsinki and Uusimaa, and a written informed consent was obtained from the participants. The experiment was conducted at the Brain and Work Research Centre of the Finnish Institute of Occupational Health.

### Peripheral blood mononuclear cells, RNA isolation, and expression microarrays

Heparinized venous blood samples were collected in sitting position from 9 participants from the experimental group and 4 participants from the control group similarly at 7∶30 AM before breakfast after BL, SR, and REC. Peripheral blood mononuclear cells (PBMC) were isolated using Ficoll density gradient centrifugation.

Total RNA was extracted by using Trizol (Gibco BRL, Paisley, UK). Final RNA concentrations were determined spectrophotometrically, and RNA quality was assessed using Agilent 2100 Bioanalyzer (Agilent Technologies, Palo Alto, CA, USA). RNA expression levels were assayed with Affymetrix GeneChip Human Genome U133 Plus 2.0 arrays (Affymetrix Inc., Santa Clara, CA, USA).

### Microarray quality control

Quality control of the expression arrays was performed with GeneSpring GX software (Agilent Technologies, Palo Alto, CA, USA). GCRMA algorithm was used for normalizing the data [64].

Following this, the Affymetrix detection calls [65] were used as a filtering criterion and probes flagged ‘present’ or ‘marginal’ in less than 2/3 of the samples were filtered out. This phase decreased the number of probes from the original 54613 to 21026.

The probes on all chips were remapped to updated Ensemble database probe set definitions (version homo_sapiens_core_54_36). After removing the un-annotated probes, 15101 probes remained (**Figure 1a**), each corresponding to a specific transcript (some genes having more than one probe).

### Transcription factor binding site analysis

Enrichment of binding sites for specific transcription factors among the up- or down-regulated genes was analyzed using the over-representation analysis tool oPOSSUM [24]. Human single site analysis was applied encompassing 5000 base pairs both upstream and downstream from the transcription start site of each gene. One-tailed Fisher exact probability test *P* values below 0.05 were considered significant.

### Pathway analysis

In order to characterize which biological pathways were enriched, a nonparametric in house developed CIGA pathway analysis program was used. This program uses Gene Ontology (GO) annotations that group the single genes in biologically meaningful pathways [25].

The analysis in the first phase consisted of all transcripts that survived probe filtering (see **Figure 1a**) comprising altogether 15101 transcripts. In order to study pathways which were activated or inactivated, the probes were divided into up- and down-regulated probes and sorted by the repeated measures ANOVA *P* value. The pathway analysis program calculates the *P* value by answering the question: “how likely is it to see this many genes (*k*) that belong to the studied pathway this high-up in the ranked list of genes (*j*), when there are altogether *t* genes that belong to the pathway amongst *n* genes in the experiment”.
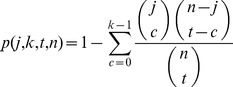



The *P* values were calculated as pointwise *P* values and permutated 1 000 times to obtain corrected *P* values. Pathways with total number of 3 genes or less were excluded from the results.

### Quantitative PCR validation of gene expression

Specific primers were ordered from Oligomer (Helsinki, Finland). Detailed primer sequences are available on request.

The cDNA was synthesized with the DyNAmo™ cDNA Synthesis Kit F-470L for qRT-PCR (Finnzymes, Helsinki, Finland) according to manufacturer's instructions. The qPCR reactions were performed as quadruplicates with DyNAmo™ Flash SYBR® Green qPCR Kit (Finnzymes) using AbiPrism 7900HT detection system (Applied Biosystems, Foster City, CA, USA). The data were analyzed using SDS software for AbiPrism 7900HT.

The relative fold difference in gene expression between SR and BL was calculated by the ΔΔCt method with qBasePLUS software (Biogazelle NV, Ghent, Belgium) taking gene specific amplification efficiencies into account [66]. The expression stability of the internal control (“housekeeping”) genes PPIA and RPLP0 was evaluated using geNormPLUS algorithm (Biogazelle) [67]. The statistical significance of the changes was assessed by paired *t* tests, and *P* values under 0.05 were considered significant.

### Interferon γ production in proliferated PBMC

Proliferation of PBMC was performed with PHA (45 µg/mL; Murex Biotech Ltd, Dartford, UK) as described in [19]. IFN-γ protein analysis was made with the Luminex bead system (Bio-Plex 200 System, Bio-Rad Laboratories, Hercules, CA, USA) by labelled cytokine capture antibody pairs (Bio-Rad Laboratories) as reported in [19]. IFN-γ protein levels in cells stimulated after SR were compared with a paired *t* test to those of cells taken from BL and stimulated similarly.

### RNA expression and CRP in the population cohort

For RNA expression analysis, the biotinylated cRNA were hybridized onto Illumina HumanHT-12 Expression BeadChips (Illumina Inc., San Diego, CA, USA) using standard protocol. For each sample, biotinylated cRNA preparation and hybridization onto BeadChip were done in duplicates [68]. Quality control and data processing were done as stated in [68]. In short, probe intensity distributions for all arrays were set to the same level by quantile normalization. Technical replicates with Pearson correlation coefficient P≥0.94 or Spearman's rank correlation coefficient ρ≥0.60 were accepted (9 samples were excluded). Linear regression model was used to correlate RNA expression with self-reported insufficient sleep adjusting for age and gender with R version 2.13.

CRP was measured from plasma using a latex immunoassay (Sentinel Diagnostics, Milan, Italy) with Architect c8000 clinical chemistry analyzer (Abbott Laboratories, Abbott Park, Illinois, US). General linear model was used for correlating CRP levels with self-reported insufficient sleep with PASW Statistics version 18 (IBM). Genders were analyzed separately (N males = 1529; N females = 1892) adjusting for age (but not for comorbidities).

### Data availability

The expression data has been made puclicly available through the ArrayExpress database (accession numbers E-MEXP-3936 and E-TABM-1036 for the experimental and population data sets, respectively).

### Ethics statement

The study was approved by the internal review board and received permission from the ethics committee of the Hospital District of Helsinki and Uusimaa, and the participants provided a written informed consent.

## Supporting Information

Table S1 Up-regulated genes after cumulative sleep restriction. List of up-regulated genes with at least 1.2-fold change after experimental sleep restriction compared to baseline and 2-way ANOVA interaction *P* value<0.05.
(DOCX)

Table S2 Down-regulated genes after cumulative sleep restriction. List of down-regulated genes with at least 1.2-fold change after experimental sleep restriction compared to baseline and 2-way ANOVA interaction *P* value<0.05.
(DOCX)

Table S3 Quantitative PCR verification of gene expression. Expression changes for six genes tested with quantitative PCR (qPCR). Five of these were verified to have a significant expression change after sleep restriction compared to baseline (four upregulated and one down-regulated as seen in the microarray (chip) data; pointwise *t* test *P*<0.05; FC  =  fold change, where BL value for each gene  = 1). One of the tested genes did not reach statistical difference in the qPCR analysis (marked with *italic*).
(DOCX)

Table S4 Biological pathways up-regulated after sleep restriction. Gene Ontology pathways (biological processes) that were significantly enriched (*P*<0.05 after permutation) among the transcripts up-regulated after sleep restriction. *Total no. of genes in pathway* represents the number of genes that are annotated to the pathway. *Top no. of genes in pathway* represents the number of genes that were found changed in the study setting and contributed to the significance of the pathway.
(DOCX)

Table S5 Biological pathways down-regulated after sleep restriction. Gene Ontology pathways (biological processes) that were significantly enriched (*P*<0.05 after permutation) among the transcripts down-regulated after sleep restriction. *Total no. of genes in pathway* represents the number of genes that are annotated to the pathway. *Top no. of genes in pathway* represents the number of genes that were found changed in the study setting and contributed to the significance of the pathway.
(DOCX)

Table S6 Toll-like receptor (TLR) coding genes up-regulated after sleep restriction. The expression levels of several transcripts coding for TLRs were up-regulated after experimental sleep restriction (SR) compared to baseline (BL) values with pointwise *t* test (P<0.05).
(DOCX)
