# Supplementary material for: Partial Sleep Restriction Activates Immune Response-Related Gene Expression Pathways: Experimental and Epidemiological Studies in Humans
Source: PLoS One. 2013 Oct 23;8(10):e77184. doi: 10.1371/journal.pone.0077184 (PMC3806729; doi:10.1371/journal.pone.0077184)
Supplement: Table S1 — Up-regulated genes after cumulative sleep restriction. List of up-regulated genes with at least 1.2-fold change after experimental sleep restriction compared to baseline and 2-way ANOVA interaction P value<0.05. (DOCX) [file pone.0077184.s001.docx]

**Table S1.** List of up-regulated genes with at least 1.2-fold change after experimental sleep restriction compared to baseline and 2-way ANOVA interaction *P* value<0.05.

| **Affymetrix Probe Set ID** | **Gene Symbol** | **Gene Title** |
| --- | --- | --- |
| 201044_x_at | DUSP1 | dual specificity phosphatase 1 |
| 221060_s_at | TLR4 | toll-like receptor 4 |
| 210148_at | HIPK3 | homeodomain interacting protein kinase 3 |
| 205033_s_at | DEFA1 / DEFA3 | defensin, alpha 1 / defensin, alpha 3, neutrophil-specific |
| 214590_s_at | UBE2D1 | ubiquitin-conjugating enzyme E2D 1 (UBC4/5 homolog, yeast) |
| 216901_s_at | IKZF1 | IKAROS family zinc finger 1 (Ikaros) |
| 229450_at | IFIT3 | interferon-induced protein with tetratricopeptide repeats 3 |
| 202672_s_at | ATF3 | activating transcription factor 3 |
| 1555214_a_at | CLEC7A | C-type lectin domain family 7, member A |
| 201235_s_at | BTG2 | BTG family, member 2 |
| 210773_s_at | FPR2 | formyl peptide receptor 2 |
| 221638_s_at | STX16 | syntaxin 16 |
| 33646_g_at | GM2A | GM2 ganglioside activator |
| 214786_at | MAP3K1 | mitogen-activated protein kinase kinase kinase 1 |
| 214511_x_at | FCGR1B | Fc fragment of IgG, high affinity Ib, receptor (CD64) |
| 221239_s_at | FCRL2 | Fc receptor-like 2 |
| 216950_s_at | FCGR1A | Fc fragment of IgG, high affinity Ia, receptor (CD64) |
| 229228_at | CREB5 | cAMP responsive element binding protein 5 |
| 232068_s_at | TLR4 | toll-like receptor 4 |
| 203923_s_at | CYBB | cytochrome b-245, beta polypeptide (chronic granulomatous disease) |
| 1555594_a_at | MBNL1 | muscleblind-like (Drosophila) |
| 211661_x_at | PTAFR | platelet-activating factor receptor |
| 200604_s_at | PRKAR1A | protein kinase, cAMP-dependent, regulatory, type I, alpha (tissue specific extinguisher 1) |
| 215159_s_at | NADK | NAD kinase |
| 214447_at | ETS1 | v-ets erythroblastosis virus E26 oncogene homolog 1 (avian) |
| 205321_at | EIF2S3 | eukaryotic translation initiation factor 2, subunit 3 gamma, 52kDa |
| 1553685_s_at | SP1 | Sp1 transcription factor |
| 219607_s_at | MS4A4A | membrane-spanning 4-domains, subfamily A, member 4 |
| 202874_s_at | ATP6V1C1 | ATPase, H+ transporting, lysosomal 42kDa, V1 subunit C1 |
| 227697_at | SOCS3 | suppressor of cytokine signaling 3 |
| 209131_s_at | SNAP23 | synaptosomal-associated protein, 23kDa |
| 224917_at | MIRN21 / TMEM49 | microRNA 21 |
| 211192_s_at | CD84 | CD84 molecule |
| 1552787_at | HELB | helicase (DNA) B |
| 224760_at | SP1 | Sp1 transcription factor |
| 205931_s_at | CREB5 | cAMP responsive element binding protein 5 |
| 210948_s_at | LEF1 | lymphoid enhancer-binding factor 1 |
| 214544_s_at | SNAP23 | synaptosomal-associated protein, 23kDa |
| 232387_at | AP1GBP1 | AP1 gamma subunit binding protein 1 |
| 210786_s_at | FLI1 | Friend leukemia virus integration 1 |
| 200769_s_at | MAT2A | methionine adenosyltransferase II, alpha |
| 217202_s_at | GLUL | glutamate-ammonia ligase (glutamine synthetase) |
| 210772_at | FPR2 | formyl peptide receptor 2 |
| 210992_x_at | FCGR2C | Fc fragment of IgG, low affinity IIc, receptor for (CD32) |
| 200890_s_at | SSR1 | signal sequence receptor, alpha (translocon-associated protein alpha) |
| 229075_at | SPATA5 | spermatogenesis associated 5 |
| 1555643_s_at | LILRA5 | leukocyte immunoglobulin-like receptor, subfamily A (with TM domain), member 5 |
| 200796_s_at | MCL1 | myeloid cell leukemia sequence 1 (BCL2-related) |
| 211861_x_at | CD28 | CD28 molecule |
| 216035_x_at | TCF7L2 | transcription factor 7-like 2 (T-cell specific, HMG-box) |
| 201669_s_at | MARCKS | myristoylated alanine-rich protein kinase C substrate |
| 211317_s_at | CFLAR | CASP8 and FADD-like apoptosis regulator |
| 200648_s_at | GLUL | glutamate-ammonia ligase (glutamine synthetase) |
| 241734_at | SRFBP1 | serum response factor binding protein 1 |
| 218035_s_at | RBM47 | RNA binding motif protein 47 |
| 1555745_a_at | LYZ | lysozyme (renal amyloidosis) |
| 207872_s_at | LILRA1 | leukocyte immunoglobulin-like receptor, subfamily A (with TM domain), member 1 |
| 202007_at | NID1 | nidogen 1 |
| 216804_s_at | PDLIM5 | PDZ and LIM domain 5 |
| 210042_s_at | CTSZ | cathepsin Z |
| 218748_s_at | EXOC5 | exocyst complex component 5 |
| 210872_x_at | GAS7 | growth arrest-specific 7 |
| 215838_at | LILRA5 | leukocyte immunoglobulin-like receptor, subfamily A (with TM domain), member 5 |
| 216899_s_at | SKAP2 | src kinase associated phosphoprotein 2 |
| 209306_s_at | SWAP70 | SWAP-70 protein |
| 211395_x_at | FCGR2C | Fc fragment of IgG, low affinity IIc, receptor for (CD32) |
| 208650_s_at | CD24 | CD24 molecule |
| 1555639_a_at | RBM14 | RNA binding motif protein 14 |
| 206920_s_at | GLE1 | GLE1 RNA export mediator homolog (yeast) |
| 217208_s_at | DLG1 | discs, large homolog 1 (Drosophila) |
| 205770_at | GSR | glutathione reductase |
| 231933_at | MARCH8 | membrane-associated ring finger (C3HC4) 8 |
| 1552691_at | ARL11 | ADP-ribosylation factor-like 11 |
| 206359_at | SOCS3 | suppressor of cytokine signaling 3 |
| 1554508_at | PIK3AP1 | phosphoinositide-3-kinase adaptor protein 1 |
| 201971_s_at | ATP6V1A | ATPase, H+ transporting, lysosomal 70kDa, V1 subunit A |
| 201299_s_at | MOBKL1B | MOB1, Mps One Binder kinase activator-like 1B (yeast) |
| 204972_at | OAS2 | 2'-5'-oligoadenylate synthetase 2, 69/71kDa |
| 213286_at | ZFR | zinc finger RNA binding protein |
| 231955_s_at | HIBADH | 3-hydroxyisobutyrate dehydrogenase |
| 238421_at | RC3H2 | ring finger and CCCH-type zinc finger domains 2 |
| 210818_s_at | BACH1 | BTB and CNC homology 1, basic leucine zipper transcription factor 1 |
| 1556035_s_at | ZNF207 | zinc finger protein 207 |
| 219073_s_at | OSBPL10 | oxysterol binding protein-like 10 |
| 209307_at | SWAP70 | SWAP-70 protein |
| 211300_s_at | TP53 | tumor protein p53 |
| 225265_at | RBMS1 | RNA binding motif, single stranded interacting protein 1 |
| 208488_s_at | CR1 | complement component (3b/4b) receptor 1 (Knops blood group) |
| 223062_s_at | PSAT1 | phosphoserine aminotransferase 1 |
| 206925_at | ST8SIA4 | ST8 alpha-N-acetyl-neuraminide alpha-2,8-sialyltransferase 4 |
| 203834_s_at | TGOLN2 | trans-golgi network protein 2 |
| 210756_s_at | NOTCH2 | Notch homolog 2 (Drosophila) |
| 204628_s_at | ITGB3 | integrin, beta 3 (platelet glycoprotein IIIa, antigen CD61) |
| 243999_at | SLFN5 | schlafen family member 5 |
| 202901_x_at | CTSS | cathepsin S |
| 208967_s_at | AK2 | adenylate kinase 2 |
| 210564_x_at | CFLAR | CASP8 and FADD-like apoptosis regulator |
| 203676_at | GNS | glucosamine (N-acetyl)-6-sulfatase (Sanfilippo disease IIID) |
| 201364_s_at | OAZ2 | ornithine decarboxylase antizyme 2 |
| 235678_at | GM2A | GM2 ganglioside activator |
| 222644_s_at | GLT25D1 | glycosyltransferase 25 domain containing 1 |
| 209795_at | CD69 | CD69 molecule |
| 232591_s_at | TMEM30A | transmembrane protein 30A |
| 201297_s_at | MOBKL1B | MOB1, Mps One Binder kinase activator-like 1B (yeast) |
| 200947_s_at | GLUD1 | glutamate dehydrogenase 1 |
| 223405_at | NPL | N-acetylneuraminate pyruvate lyase (dihydrodipicolinate synthase) |
| 239630_at | TAS2R14 | Taste receptor type 2 member 14 |
| 214697_s_at | ROD1 | ROD1 regulator of differentiation 1 (S. pombe) |
| 205254_x_at | TCF7 | transcription factor 7 (T-cell specific, HMG-box) |
| 222837_s_at | NARG1 | NMDA receptor regulated 1 |
| 211862_x_at | CFLAR | CASP8 and FADD-like apoptosis regulator |
| 242458_at | RALGPS2 | Ral GEF with PH domain and SH3 binding motif 2 |
| 212762_s_at | TCF7L2 | transcription factor 7-like 2 (T-cell specific, HMG-box) |
| 204924_at | TLR2 | toll-like receptor 2 |
| 207091_at | P2RX7 | purinergic receptor P2X, ligand-gated ion channel, 7 |
| 1553134_s_at | C9orf72 | chromosome 9 open reading frame 72 |
| 208485_x_at | CFLAR | CASP8 and FADD-like apoptosis regulator |
| 203300_x_at | AP1S2 | adaptor-related protein complex 1, sigma 2 subunit |
| 210873_x_at | APOBEC3A | apolipoprotein B mRNA editing enzyme, catalytic polypeptide-like 3A |
| 202008_s_at | NID1 | nidogen 1 |
| 211825_s_at | EWSR1 / FLI1 | Ewing sarcoma breakpoint region 1 / Friend leukemia virus integration 1 |
| 222849_s_at | SCRN3 | secernin 3 |
| 203005_at | LTBR | lymphotoxin beta receptor (TNFR superfamily, member 3) |
| 200742_s_at | TPP1 | tripeptidyl peptidase I |
| 212529_at | LSM12 | LSM12 homolog (S. cerevisiae) |
| 222502_s_at | UFM1 | ubiquitin-fold modifier 1 |
| 1555565_s_at | TAPBP | TAP binding protein (tapasin) |
| 221039_s_at | DDEF1 | development and differentiation enhancing factor 1 |
| 238458_at | EFHA2 | EF-hand domain family, member A2 |
| 208917_x_at | NADK | NAD kinase |
| 201151_s_at | MBNL1 | muscleblind-like (Drosophila) |
| 203242_s_at | PDLIM5 | PDZ and LIM domain 5 |
| 224358_s_at | MS4A7 | membrane-spanning 4-domains, subfamily A, member 7 |
| 209906_at | C3AR1 | complement component 3a receptor 1 |
| 1554481_a_at | EPB41 | erythrocyte membrane protein band 4.1 (elliptocytosis 1, RH-linked) |
| 46947_at | GNL3L | guanine nucleotide binding protein-like 3 (nucleolar)-like |
| 200889_s_at | SSR1 | signal sequence receptor, alpha (translocon-associated protein alpha) |
| 218832_x_at | ARRB1 | arrestin, beta 1 |
| 216243_s_at | IL1RN | interleukin 1 receptor antagonist |
| 210379_s_at | TLK1 | tousled-like kinase 1 |
| 220990_s_at | MIRN21 / TMEM49 | transmembrane protein 49 / microRNA 21 |
| 211672_s_at | ARPC4 / TTLL3 | actin related protein 2/3 complex, subunit 4, 20kDa / tubulin tyrosine ligase-like family, member 3 |
| 205026_at | STAT5B | signal transducer and activator of transcription 5B |
| 1554464_a_at | CRTAP | cartilage associated protein |
| 210784_x_at | LILRA6 / LILRB3 | leukocyte immunoglobulin-like receptor, subfamily B, member 3 / member 6 |
| 211102_s_at | LILRA2 | leukocyte immunoglobulin-like receptor, subfamily A, member 2 |
| 201538_s_at | DUSP3 | dual specificity phosphatase 3 |
| 1554569_a_at | CUGBP2 | CUG triplet repeat, RNA binding protein 2 |
| 204362_at | SKAP2 | src kinase associated phosphoprotein 2 |
| 242288_s_at | EMILIN2 | elastin microfibril interfacer 2 |
| 211077_s_at | TLK1 | tousled-like kinase 1 |
| 227609_at | EPSTI1 | epithelial stromal interaction 1 (breast) |
| 203543_s_at | KLF9 | Kruppel-like factor 9 |
| 207104_x_at | LILRB1 | leukocyte immunoglobulin-like receptor, subfamily B (with TM and ITIM domains), member 1 |
| 212514_x_at | DDX3X | DEAD (Asp-Glu-Ala-Asp) box polypeptide 3, X-linked |
| 1568954_s_at | C16orf72 | chromosome 16 open reading frame 72 |
| 201298_s_at | MOBKL1B | MOB1, Mps One Binder kinase activator-like 1B (yeast) |
| 200008_s_at | GDI2 | GDP dissociation inhibitor 2 |
| 228055_at | NAPSB | napsin B aspartic peptidase pseudogene |
| 224407_s_at | RP6-213H19.1 | serine/threonine protein kinase MST4 |
| 212911_at | DNAJC16 | DnaJ (Hsp40) homolog, subfamily C, member 16 |
| 202609_at | EPS8 | epidermal growth factor receptor pathway substrate 8 |
| 202430_s_at | PLSCR1 | phospholipid scramblase 1 |
| 202944_at | NAGA | N-acetylgalactosaminidase, alpha- |
| 1565717_s_at | FUS | fusion (involved in t(12;16) in malignant liposarcoma) |
| 201237_at | CAPZA2 | capping protein (actin filament) muscle Z-line, alpha 2 |
| 214336_s_at | COPA | coatomer protein complex, subunit alpha |
| 209004_s_at | FBXL5 | F-box and leucine-rich repeat protein 5 |
| 216037_x_at | TCF7L2 | transcription factor 7-like 2 (T-cell specific, HMG-box) |
| 218231_at | NAGK | N-acetylglucosamine kinase |
| 227066_at | MOBKL2C | MOB1, Mps One Binder kinase activator-like 2C (yeast) |
| 210371_s_at | RBBP4 | retinoblastoma binding protein 4 |
| 205851_at | NME6 | non-metastatic cells 6, protein expressed in (nucleoside-diphosphate kinase) |
| 201537_s_at | DUSP3 | dual specificity phosphatase 3 |
| 225837_at | C12orf32 | chromosome 12 open reading frame 32 |
| 214486_x_at | CFLAR | CASP8 and FADD-like apoptosis regulator |
| 208653_s_at | CD164 | CD164 molecule, sialomucin |
| 235574_at | GBP4 | guanylate binding protein 4 |
| 217301_x_at | RBBP4 | retinoblastoma binding protein 4 |
| 226702_at | CMPK2 | cytidine monophosphate (UMP-CMP) kinase 2, mitochondrial |
| 209508_x_at | CFLAR | CASP8 and FADD-like apoptosis regulator |
| 200605_s_at | PRKAR1A | protein kinase, cAMP-dependent, regulatory, type I, alpha (tissue specific extinguisher 1) |
| 207350_s_at | VAMP4 | vesicle-associated membrane protein 4 |
| 242064_at | SDK2 | sidekick homolog 2 (chicken) |
| 225662_at | ZAK | sterile alpha motif and leucine zipper containing kinase AZK |
| 1555797_a_at | ARPC5 | actin related protein 2/3 complex, subunit 5, 16kDa |
| 225604_s_at | C9orf19 | chromosome 9 open reading frame 19 |
| 209761_s_at | SP110 | SP110 nuclear body protein |
| 208875_s_at | PAK2 | p21 (CDKN1A)-activated kinase 2 |
| 214196_s_at | TPP1 | tripeptidyl peptidase I |
| 212820_at | DMXL2 | Dmx-like 2 |
| 206133_at | XAF1 | XIAP associated factor 1 |
| 225290_at | ETNK1 | ethanolamine kinase 1 |
| 201614_s_at | RUVBL1 | RuvB-like 1 (E. coli) |
| 1555756_a_at | CLEC7A | C-type lectin domain family 7, member A |
| 225045_at | CCDC88A | coiled-coil domain containing 88A |
| 219165_at | PDLIM2 | PDZ and LIM domain 2 (mystique) |
| 1552613_s_at | CDC42SE2 | CDC42 small effector 2 |
| 200641_s_at | YWHAZ | tyrosine 3-monooxygenase/tryptophan 5-monooxygenase activation protein, zeta polypeptide |
| 209046_s_at | GABARAPL2 | GABA(A) receptor-associated protein-like 2 |
| 1554152_a_at | OGDH | oxoglutarate (alpha-ketoglutarate) dehydrogenase (lipoamide) |
| 239346_at | GTF2H3 | Transcribed locus, moderately similar to XP_001713735.1 |
| 210458_s_at | TANK | TRAF family member-associated NFKB activator |
| 217294_s_at | ENO1 | enolase 1, (alpha) |
| 201238_s_at | CAPZA2 | capping protein (actin filament) muscle Z-line, alpha 2 |
| 238461_at | EIF4E3 | eukaryotic translation initiation factor 4E family member 3 |
| 212398_at | RDX | radixin |
| 218152_at | HMG20A | high-mobility group 20A |
| 204439_at | IFI44L | interferon-induced protein 44-like |
| 231817_at | USP53 | ubiquitin specific peptidase 53 |
| 202833_s_at | SERPINA1 | serpin peptidase inhibitor, clade A (alpha-1 antiproteinase, antitrypsin), member 1 |
| 216202_s_at | SPTLC2 | serine palmitoyltransferase, long chain base subunit 2 |
| 223131_s_at | TRIM8 | tripartite motif-containing 8 |
| 206360_s_at | SOCS3 | suppressor of cytokine signaling 3 |
| 202069_s_at | IDH3A | isocitrate dehydrogenase 3 (NAD+) alpha |
| 226388_at | TCEA3 | transcription elongation factor A (SII), 3 |
| 1554414_a_at | OSGIN2 | oxidative stress induced growth inhibitor family member 2 |
| 201780_s_at | RNF13 | ring finger protein 13 |
| 217552_x_at | CR1 | complement component (3b/4b) receptor 1 (Knops blood group) |
| 235798_at | TMEM170B | hypothetical LOC100113407 |
| 210563_x_at | CFLAR | CASP8 and FADD-like apoptosis regulator |
| 228867_at | TATDN3 | TatD DNase domain containing 3 |
| 209930_s_at | NFE2 | nuclear factor (erythroid-derived 2), 45kDa |
| 211546_x_at | SNCA | synuclein, alpha (non A4 component of amyloid precursor) |
| 203140_at | BCL6 | B-cell CLL/lymphoma 6 (zinc finger protein 51) |
| 1558279_a_at | KDSR | 3-ketodihydrosphingosine reductase |
