# Supplementary material for: Partial Sleep Restriction Activates Immune Response-Related Gene Expression Pathways: Experimental and Epidemiological Studies in Humans
Source: PLoS One. 2013 Oct 23;8(10):e77184. doi: 10.1371/journal.pone.0077184 (PMC3806729; doi:10.1371/journal.pone.0077184)
Supplement: Table S2 — Down-regulated genes after cumulative sleep restriction. List of down-regulated genes with at least 1.2-fold change after experimental sleep restriction compared to baseline and 2-way ANOVA interaction P value<0.05. (DOCX) [file pone.0077184.s002.docx]

**Table S2.** List of down-regulated genes with at least 1.2-fold change after experimental sleep restriction compared to baseline and 2-way ANOVA interaction *P* value<0.05.

| **Affymetrix Probe Set ID** | **Gene Symbol** | **Gene Title** |
| --- | --- | --- |
| 211687_x_at | KIR3DL1 | killer cell immunoglobulin-like receptor, three domains, long cytoplasmic tail, 1 |
| 210164_at | GZMB | granzyme B (granzyme 2, cytotoxic T-lymphocyte-associated serine esterase 1) |
| 212843_at | NCAM1 | neural cell adhesion molecule 1 |
| 228774_at | CEP78 | centrosomal protein 78kDa |
| 210321_at | GZMH | granzyme H (cathepsin G-like 2, protein h-CCPX) |
| 220646_s_at | KLRF1 | killer cell lectin-like receptor subfamily F, member 1 |
| 227819_at | LGR6 | leucine-rich repeat-containing G protein-coupled receptor 6 |
| 228063_s_at | NAP1L5 | nucleosome assembly protein 1-like 5 |
| 37145_at | GNLY | granulysin |
| 232914_s_at | SYTL2 | synaptotagmin-like 2 |
| 205495_s_at | GNLY | granulysin |
| 207072_at | IL18RAP | interleukin 18 receptor accessory protein |
| 226625_at | TGFBR3 | transforming growth factor, beta receptor III |
| 226858_at | CSNK1E | casein kinase 1, epsilon |
| 210140_at | CST7 | cystatin F (leukocystatin) |
| 220684_at | TBX21 | T-box 21 |
| 227394_at | NCAM1 | neural cell adhesion molecule 1 |
| 213915_at | NKG7 | natural killer cell group 7 sequence |
| 206267_s_at | MATK | megakaryocyte-associated tyrosine kinase |
| 202146_at | IFRD1 | interferon-related developmental regulator 1 |
| 205291_at | IL2RB | interleukin 2 receptor, beta |
| 204275_at | SOLH | small optic lobes homolog (Drosophila) |
| 209993_at | ABCB1 | ATP-binding cassette, sub-family B (MDR/TAP), member 1 |
| 1553736_at | CCDC131 | coiled-coil domain containing 131 |
| 214450_at | CTSW | cathepsin W |
| 235232_at | GMEB1 | glucocorticoid modulatory element binding protein 1 |
| 214470_at | KLRB1 | killer cell lectin-like receptor subfamily B, member 1 |
| 224315_at | DDX20 | DEAD (Asp-Glu-Ala-Asp) box polypeptide 20 |
| 213996_at | YPEL1 | yippee-like 1 (Drosophila) |
| 228667_at | AGPAT4 | 1-acylglycerol-3-phosphate O-acyltransferase 4 (lysophosphatidic acid acyltransferase, delta) |
| 223543_at | PDZD4 | PDZ domain containing 4 |
| 225099_at | FBXO45 | F-box protein 45 |
| 221216_s_at | SCMH1 | sex comb on midleg homolog 1 (Drosophila) |
| 206724_at | CBX4 | chromobox homolog 4 (Pc class homolog, Drosophila) |
| 211840_s_at | PDE4D | phosphodiesterase 4D, cAMP-specific (phosphodiesterase E3 dunce homolog, Drosophila) |
| 209994_s_at | ABCB1 / ABCB4 | ATP-binding cassette, sub-family B (MDR/TAP), member 1 / member 4 |
| 213787_s_at | EBP | emopamil binding protein (sterol isomerase) |
| 207460_at | GZMM | granzyme M (lymphocyte met-ase 1) |
| 209197_at | SYT11 | synaptotagmin XI |
| 222870_s_at | B3GNT2 | UDP-GlcNAc:betaGal beta-1,3-N-acetylglucosaminyltransferase 2 |
| 212772_s_at | ABCA2 | ATP-binding cassette, sub-family A (ABC1), member 2 |
| 204529_s_at | TOX | thymocyte selection-associated high mobility group box |
| 212333_at | FAM98A | family with sequence similarity 98, member A |
| 227650_at | HSPA14 | heat shock 70kDa protein 14 |
| 226689_at | CISD2 | CDGSH iron sulfur domain 2 |
| 242905_at | PNO1 | partner of NOB1 homolog (S. cerevisiae) |
| 202524_s_at | SPOCK2 | sparc/osteonectin, cwcv and kazal-like domains proteoglycan (testican) 2 |
| 222808_at | ALG13 / CXorf45 | asparagine-linked glycosylation 13 homolog (S. cerevisiae) |
| 201745_at | TWF1 | twinfilin, actin-binding protein, homolog 1 (Drosophila) |
| 200733_s_at | PTP4A1 | protein tyrosine phosphatase type IVA, member 1 |
| 204006_s_at | FCGR3A / FCGR3B | Fc fragment of IgG, low affinity IIIa / IIIb, receptor (CD16a/CD16b) |
| 209524_at | HDGFRP3 | hepatoma-derived growth factor, related protein 3 |
| 229963_at | BEX5 | BEX family member 5 |
| 242463_x_at | ZNF600 | zinc finger protein 600 |
| 213372_at | PAQR3 | progestin and adipoQ receptor family member III |
| 222801_s_at | STAG3L4 | stromal antigen 3-like 4 |
| 213954_at | KIAA0888 | KIAA0888 protein |
| 219117_s_at | FKBP11 | FK506 binding protein 11, 19 kDa |
| 224992_s_at | CMIP | c-Maf-inducing protein |
| 207357_s_at | GALNT10 | UDP-N-acetyl-alpha-D-galactosamine:polypeptide N-acetylgalactosaminyltransferase 10 |
| 223514_at | CARD11 | caspase recruitment domain family, member 11 |
| 212742_at | ZNF364 | zinc finger protein 364 |
| 211144_x_at | TARP / TRGC2 | T cell receptor gamma constant 2 / TCR gamma alternate reading frame protein |
| 210053_at | TAF5 | TAF5 RNA polymerase II, TATA box binding protein (TBP)-associated factor, 100kDa |
| 225747_at | COQ10A | coenzyme Q10 homolog A (S. cerevisiae) |
| 202725_at | POLR2A | polymerase (RNA) II (DNA directed) polypeptide A, 220kDa |
| 219347_at | NUDT15 | nudix (nucleoside diphosphate linked moiety X)-type motif 15 |
| 1555325_s_at | ZNF26 | zinc finger protein 26 |
| 219878_s_at | KLF13 | Kruppel-like factor 13 |
| 224576_at | ERGIC1 | endoplasmic reticulum-golgi intermediate compartment (ERGIC) 1 |
| 230760_at | ZFY | zinc finger protein, Y-linked / hypothetical protein LOC100130829 |
| 210361_s_at | ELF2 | E74-like factor 2 (ets domain transcription factor) |
| 223370_at | PLEKHA3 | pleckstrin homology domain containing, family A (phosphoinositide binding specific) member 3 |
| 216033_s_at | FYN | FYN oncogene related to SRC, FGR, YES |
| 200710_at | ACADVL | acyl-Coenzyme A dehydrogenase, very long chain |
| 212098_at | LOC151162 | hypothetical LOC151162 |
| 202149_at | NEDD9 | neural precursor cell expressed, developmentally down-regulated 9 |
| 235020_at | TAF4B | TAF4b RNA polymerase II, TATA box binding protein (TBP)-associated factor, 105kDa |
| 221335_x_at | C19orf61 | chromosome 19 open reading frame 61 |
| 201701_s_at | PGRMC2 | progesterone receptor membrane component 2 |
| 234165_at | PTGDR | prostaglandin D2 receptor (DP) |
| 1554016_a_at | C16orf57 | chromosome 16 open reading frame 57 |
| 219797_at | MGAT4A | mannosyl (alpha-1,3-)-glycoprotein beta-1,4-N-acetylglucosaminyltransferase, isozyme A |
