# Supplementary material for: Partial Sleep Restriction Activates Immune Response-Related Gene Expression Pathways: Experimental and Epidemiological Studies in Humans
Source: PLoS One. 2013 Oct 23;8(10):e77184. doi: 10.1371/journal.pone.0077184 (PMC3806729; doi:10.1371/journal.pone.0077184)
Supplement: Table S3 — Quantitative PCR verification of gene expression. Expression changes for six genes tested with quantitative PCR (qPCR). Five of these were verified to have a significant expression change after sleep restriction compared to baseline (four upregulated and one down-regulated as seen in the microarray (chip) data; pointwise t test P<0.05; FC = fold change, where BL value for each gene = 1). One of the tested genes did not reach statistical difference in the qPCR analysis (marked with italic). (DOCX) [file pone.0077184.s003.docx]

**Table S3.** Expression changes for six genes tested with quantitative RT-PCR (qRT-PCR). Five of these were verified to have a significant expression change after sleep restriction compared to baseline (four up-regulated and one down-regulated as seen in the microarray (chip) data; pointwise *t* test *P*<0.05; FC = fold change, where BL value for each gene = 1). One of the tested genes did not reach statistical difference in the qRT-PCR analysis (marked with *italic*).

|  | **qRT-PCR** | | **Chip** |
| --- | --- | --- | --- |
| **Gene** | ***P* value** | **FC** | **FC** |
| TGFBR3 | 0.003 | 0.71 | 0.65 |
| BTG2 | 0.012 | 1.24 | 1.65 |
| FCRL2 | 0.013 | 1.38 | 1.58 |
| STX16 | 0.025 | 1.18 | 1.63 |
| IKZF1 | 0.046 | 1.14 | 1.71 |
| HIPK3 | *0.141* | *1.10* | 1.91 |
