# Supplementary material for: Partial Sleep Restriction Activates Immune Response-Related Gene Expression Pathways: Experimental and Epidemiological Studies in Humans
Source: PLoS One. 2013 Oct 23;8(10):e77184. doi: 10.1371/journal.pone.0077184 (PMC3806729; doi:10.1371/journal.pone.0077184)
Supplement: Table S4 — Biological pathways up-regulated after sleep restriction. Gene Ontology pathways (biological processes) that were significantly enriched (P<0.05 after permutation) among the transcripts up-regulated after sleep restriction. Total no. of genes in pathway represents the number of genes that are annotated to the pathway. Top no. of genes in pathway represents the number of genes that were found changed in the study setting and contributed to the significance of the pathway. (DOCX) [file pone.0077184.s004.docx]

**Table S4.** Gene Ontology pathways (biological processes) that were significantly enriched (*P*<0.05 after permutation) among the transcripts up-regulated after sleep restriction. *Total no. of genes in pathway* represents the number of genes that are annotated to the pathway. *Top no. of genes in pathway* represents the number of genes that were found in the study setting and contributed to the significance of the pathway.

| **Gene Ontology ID** | **Pathway** | **Opt. *P* value** | **Permuted *P* value** | **Total no. of genes in pathway** | **Gene rank** | **Top no. of genes in pathway** |
| --- | --- | --- | --- | --- | --- | --- |
| GO:0042113 | B cell activation | 3.62E-06 | 0.001 | 90 | 1868 | 43 |
| GO:0032637 | interleukin-8 production | 6.49E-06 | 0.001 | 9 | 1282 | 8 |
| GO:0001530 | lipopolysaccharide binding | 6.49E-06 | 0.001 | 9 | 1282 | 8 |
| GO:0006805 | xenobiotic metabolic process | 1.23E-05 | 0.001 | 8 | 352 | 5 |
| GO:0050817 | coagulation | 1.40E-05 | 0.001 | 75 | 821 | 22 |
| GO:0045321 | leukocyte activation | 1.77E-05 | 0.001 | 208 | 1499 | 68 |
| GO:0001775 | cell activation | 2.20E-05 | 0.002 | 217 | 1499 | 70 |
| GO:0002460 | adaptive immune response based on somatic recombination of immune receptors built from immunoglobulin superfamily domains | 2.41E-05 | 0.002 | 83 | 2853 | 51 |
| GO:0005543 | phospholipid binding | 2.63E-05 | 0.003 | 91 | 1083 | 29 |
| GO:0046649 | lymphocyte activation | 2.96E-05 | 0.002 | 184 | 1499 | 61 |
| GO:0045416 | positive regulation of interleukin-8 biosynthetic process | 3.32E-05 | 0.001 | 8 | 1282 | 7 |
| GO:0042228 | interleukin-8 biosynthetic process | 3.32E-05 | 0.001 | 8 | 1282 | 7 |
| GO:0045414 | regulation of interleukin-8 biosynthetic process | 3.32E-05 | 0.001 | 8 | 1282 | 7 |
| GO:0015671 | oxygen transport | 3.61E-05 | 0.001 | 10 | 1329 | 8 |
| GO:0005833 | hemoglobin complex | 3.61E-05 | 0.001 | 10 | 1329 | 8 |
| GO:0002250 | adaptive immune response | 3.74E-05 | 0.001 | 88 | 2853 | 53 |
| GO:0002521 | leukocyte differentiation | 3.89E-05 | 0.002 | 110 | 2624 | 60 |
| GO:0009410 | response to xenobiotic stimulus | 5.10E-05 | 0.003 | 10 | 352 | 5 |
| GO:0005885 | Arp2/3 protein complex | 6.33E-05 | 0.001 | 15 | 3855 | 15 |
| GO:0030098 | lymphocyte differentiation | 7.09E-05 | 0.005 | 93 | 1482 | 35 |
| GO:0007249 | I-kappaB kinase/NF-kappaB cascade | 8.34E-05 | 0.003 | 107 | 1499 | 39 |
| GO:0042108 | positive regulation of cytokine biosynthetic process | 9.67E-05 | 0.003 | 26 | 2593 | 19 |
| GO:0015669 | gas transport | 1.11E-04 | 0.001 | 11 | 1329 | 8 |
| GO:0007596 | blood coagulation | 1.44E-04 | 0.006 | 65 | 588 | 15 |
| GO:0009620 | response to fungus | 1.51E-04 | 0.001 | 17 | 1282 | 10 |
| GO:0002695 | negative regulation of leukocyte activation | 1.88E-04 | 0.001 | 19 | 907 | 9 |
| GO:0050866 | negative regulation of cell activation | 1.88E-04 | 0.002 | 19 | 907 | 9 |
| GO:0042094 | interleukin-2 biosynthetic process | 1.95E-04 | 0.001 | 9 | 2843 | 9 |
| GO:0002449 | lymphocyte mediated immunity | 2.08E-04 | 0.007 | 76 | 3212 | 49 |
| GO:0045086 | positive regulation of interleukin-2 biosynthetic process | 2.41E-04 | 0.002 | 8 | 2593 | 8 |
| GO:0045076 | regulation of interleukin-2 biosynthetic process | 2.41E-04 | 0.002 | 8 | 2593 | 8 |
| GO:0050792 | regulation of viral reproduction | 2.41E-04 | 0.003 | 8 | 2593 | 8 |
| GO:0030097 | hemopoiesis | 2.45E-04 | 0.011 | 160 | 2624 | 79 |
| GO:0050819 | negative regulation of coagulation | 2.47E-04 | 0.001 | 10 | 807 | 6 |
| GO:0004859 | phospholipase inhibitor activity | 2.47E-04 | 0.002 | 10 | 807 | 6 |
| GO:0055102 | lipase inhibitor activity | 2.47E-04 | 0.005 | 10 | 807 | 6 |
| GO:0002520 | immune system development | 2.59E-04 | 0.013 | 176 | 2718 | 88 |
| GO:0030856 | regulation of epithelial cell differentiation | 2.76E-04 | 0.003 | 9 | 2954 | 9 |
| GO:0045884 | regulation of survival gene product expression | 2.93E-04 | 0.002 | 13 | 2528 | 11 |
| GO:0006916 | anti-apoptosis | 2.99E-04 | 0.006 | 153 | 1933 | 60 |
| GO:0002443 | leukocyte mediated immunity | 3.00E-04 | 0.012 | 85 | 2599 | 46 |
| GO:0001848 | complement binding | 3.11E-04 | 0.002 | 8 | 1152 | 6 |
| GO:0050850 | positive regulation of calcium-mediated signaling | 3.37E-04 | 0.002 | 12 | 2954 | 11 |
| GO:0050848 | regulation of calcium-mediated signaling | 3.37E-04 | 0.005 | 12 | 2954 | 11 |
| GO:0009595 | detection of biotic stimulus | 3.59E-04 | 0.005 | 13 | 2579 | 11 |
| GO:0045620 | negative regulation of lymphocyte differentiation | 3.67E-04 | 0.003 | 8 | 367 | 4 |
| GO:0045581 | negative regulation of T cell differentiation | 3.67E-04 | 0.004 | 8 | 367 | 4 |
| GO:0045637 | regulation of myeloid cell differentiation | 3.76E-04 | 0.004 | 25 | 2706 | 18 |
| GO:0006954 | inflammatory response | 3.79E-04 | 0.007 | 169 | 1160 | 44 |
| GO:0033764 | steroid dehydrogenase activity. acting on the CH-OH group of donors. NAD or NADP as acceptor | 3.85E-04 | 0.003 | 9 | 3066 | 9 |
| GO:0001816 | cytokine production | 4.04E-04 | 0.015 | 89 | 1160 | 27 |
| GO:0050868 | negative regulation of T cell activation | 4.43E-04 | 0.005 | 18 | 439 | 6 |
| GO:0051250 | negative regulation of lymphocyte activation | 4.43E-04 | 0.007 | 18 | 439 | 6 |
| GO:0048546 | digestive tract morphogenesis | 4.47E-04 | 0.003 | 8 | 2800 | 8 |
| GO:0048547 | gut morphogenesis | 4.47E-04 | 0.005 | 8 | 2800 | 8 |
| GO:0050776 | regulation of immune response | 4.66E-04 | 0.015 | 91 | 3215 | 56 |
| GO:0007599 | hemostasis | 4.73E-04 | 0.013 | 72 | 588 | 15 |
| GO:0009952 | anterior/posterior pattern formation | 4.93E-04 | 0.01 | 34 | 34 | 3 |
| GO:0030183 | B cell differentiation | 4.99E-04 | 0.011 | 53 | 1868 | 25 |
| GO:0045619 | regulation of lymphocyte differentiation | 5.18E-04 | 0.01 | 45 | 1339 | 18 |
| GO:0002682 | regulation of immune system process | 5.35E-04 | 0.015 | 158 | 3215 | 90 |
| GO:0004499 | flavin-containing monooxygenase activity | 5.57E-04 | 0.005 | 16 | 748 | 7 |
| GO:0050878 | regulation of body fluid levels | 6.43E-04 | 0.016 | 74 | 588 | 15 |
| GO:0006891 | intra-Golgi vesicle-mediated transport | 6.51E-04 | 0.004 | 15 | 1435 | 9 |
| GO:0048534 | hemopoietic or lymphoid organ development | 6.72E-04 | 0.017 | 166 | 2718 | 82 |
| GO:0006690 | icosanoid metabolic process | 6.80E-04 | 0.01 | 26 | 2948 | 19 |
| GO:0003002 | regionalization | 7.41E-04 | 0.014 | 39 | 34 | 3 |
| GO:0016064 | immunoglobulin mediated immune response | 7.59E-04 | 0.015 | 50 | 2853 | 31 |
| GO:0019724 | B cell mediated immunity | 7.59E-04 | 0.022 | 50 | 2853 | 31 |
| GO:0045580 | regulation of T cell differentiation | 7.73E-04 | 0.008 | 36 | 527 | 9 |
| GO:0042060 | wound healing | 8.02E-04 | 0.016 | 92 | 478 | 15 |
| GO:0046696 | lipopolysaccharide receptor complex | 8.29E-04 | 0.005 | 6 | 1282 | 5 |
| GO:0031201 | SNARE complex | 8.61E-04 | 0.007 | 6 | 1292 | 5 |
| GO:0050818 | regulation of coagulation | 8.94E-04 | 0.01 | 12 | 807 | 6 |
| GO:0032680 | regulation of tumor necrosis factor production | 9.41E-04 | 0.005 | 14 | 1282 | 8 |
| GO:0045087 | innate immune response | 9.68E-04 | 0.017 | 77 | 2599 | 41 |
| GO:0045444 | fat cell differentiation | 9.84E-04 | 0.012 | 17 | 3133 | 14 |
| GO:0003727 | single-stranded RNA binding | 1.00E-03 | 0.007 | 16 | 1094 | 8 |
| GO:0042805 | actinin binding | 1.01E-03 | 0.006 | 7 | 565 | 4 |
| GO:0002683 | negative regulation of immune system process | 1.06E-03 | 0.017 | 32 | 907 | 11 |
| GO:0016229 | steroid dehydrogenase activity | 1.10E-03 | 0.007 | 11 | 3066 | 10 |
| GO:0045638 | negative regulation of myeloid cell differentiation | 1.10E-03 | 0.009 | 11 | 1339 | 7 |
| GO:0019825 | oxygen binding | 1.21E-03 | 0.011 | 14 | 1329 | 8 |
| GO:0043123 | positive regulation of I-kappaB kinase/NF-kappaB cascade | 1.23E-03 | 0.03 | 75 | 1499 | 27 |
| GO:0016046 | detection of fungus | 1.31E-03 | 0.008 | 8 | 2208 | 7 |
| GO:0048562 | embryonic organ morphogenesis | 1.33E-03 | 0.011 | 11 | 1865 | 8 |
| GO:0007389 | pattern specification process | 1.33E-03 | 0.037 | 63 | 448 | 11 |
| GO:0001818 | negative regulation of cytokine production | 1.34E-03 | 0.01 | 8 | 3212 | 8 |
| GO:0030217 | T cell differentiation | 1.36E-03 | 0.031 | 57 | 1210 | 19 |
| GO:0042093 | T-helper cell differentiation | 1.37E-03 | 0.006 | 8 | 222 | 3 |
| GO:0045628 | regulation of T-helper 2 cell differentiation | 1.37E-03 | 0.009 | 8 | 222 | 3 |
| GO:0002294 | CD4-positive. alpha-beta T cell differentiation during immune response | 1.37E-03 | 0.01 | 8 | 222 | 3 |
| GO:0002292 | T cell differentiation during immune response | 1.37E-03 | 0.011 | 8 | 222 | 3 |
| GO:0045622 | regulation of T-helper cell differentiation | 1.37E-03 | 0.011 | 8 | 222 | 3 |
| GO:0002293 | alpha-beta T cell differentiation during immune response | 1.37E-03 | 0.011 | 8 | 222 | 3 |
| GO:0002285 | lymphocyte activation during immune response | 1.37E-03 | 0.013 | 8 | 222 | 3 |
| GO:0045064 | T-helper 2 cell differentiation | 1.37E-03 | 0.013 | 8 | 222 | 3 |
| GO:0002286 | T cell activation during immune response | 1.37E-03 | 0.014 | 8 | 222 | 3 |
| GO:0051241 | negative regulation of multicellular organismal process | 1.42E-03 | 0.019 | 33 | 907 | 11 |
| GO:0005544 | calcium-dependent phospholipid binding | 1.42E-03 | 0.02 | 15 | 942 | 7 |
| GO:0002684 | positive regulation of immune system process | 1.60E-03 | 0.037 | 126 | 3212 | 72 |
| GO:0050778 | positive regulation of immune response | 1.71E-03 | 0.034 | 79 | 2853 | 44 |
| GO:0051606 | detection of stimulus | 1.72E-03 | 0.026 | 32 | 1282 | 13 |
| GO:0046006 | regulation of activated T cell proliferation | 1.76E-03 | 0.009 | 10 | 2954 | 9 |
| GO:0042104 | positive regulation of activated T cell proliferation | 1.76E-03 | 0.018 | 10 | 2954 | 9 |
| GO:0005344 | oxygen transporter activity | 1.78E-03 | 0.006 | 9 | 1329 | 6 |
| GO:0050864 | regulation of B cell activation | 1.78E-03 | 0.019 | 31 | 2954 | 21 |
| GO:0045646 | regulation of erythrocyte differentiation | 1.80E-03 | 0.016 | 14 | 2706 | 11 |
| GO:0045066 | regulatory T cell differentiation | 1.94E-03 | 0.008 | 6 | 2593 | 6 |
| GO:0045069 | regulation of viral genome replication | 1.94E-03 | 0.016 | 6 | 2593 | 6 |
| GO:0016628 | oxidoreductase activity. acting on the CH-CH group of donors. NAD or NADP as acceptor | 1.99E-03 | 0.015 | 23 | 299 | 5 |
| GO:0034101 | erythrocyte homeostasis | 2.00E-03 | 0.029 | 33 | 3891 | 26 |
| GO:0002263 | cell activation during immune response | 2.00E-03 | 0.009 | 9 | 222 | 3 |
| GO:0043370 | regulation of CD4-positive. alpha beta T cell differentiation | 2.00E-03 | 0.013 | 9 | 222 | 3 |
| GO:0043367 | CD4-positive. alpha beta T cell differentiation | 2.00E-03 | 0.016 | 9 | 222 | 3 |
| GO:0002366 | leukocyte activation during immune response | 2.00E-03 | 0.019 | 9 | 222 | 3 |
| GO:0043029 | T cell homeostasis | 2.04E-03 | 0.015 | 15 | 2954 | 12 |
| GO:0043627 | response to estrogen stimulus | 2.29E-03 | 0.029 | 23 | 3122 | 17 |
| GO:0006693 | prostaglandin metabolic process | 2.30E-03 | 0.016 | 14 | 3325 | 12 |
| GO:0006692 | prostanoid metabolic process | 2.30E-03 | 0.023 | 14 | 3325 | 12 |
| GO:0008081 | phosphoric diester hydrolase activity | 2.38E-03 | 0.027 | 23 | 185 | 4 |
| GO:0050863 | regulation of T cell activation | 2.38E-03 | 0.041 | 75 | 527 | 13 |
| GO:0019864 | IgG binding | 2.38E-03 | 0.012 | 7 | 709 | 4 |
| GO:0043122 | regulation of I-kappaB kinase/NF-kappaB cascade | 2.39E-03 | 0.045 | 78 | 1499 | 27 |
| GO:0035091 | phosphoinositide binding | 2.42E-03 | 0.036 | 66 | 2393 | 33 |
| GO:0008637 | apoptotic mitochondrial changes | 2.44E-03 | 0.021 | 20 | 2348 | 13 |
| GO:0007156 | homophilic cell adhesion | 2.52E-03 | 0.025 | 16 | 961 | 7 |
| GO:0002694 | regulation of leukocyte activation | 2.60E-03 | 0.033 | 88 | 3212 | 52 |
| GO:0016709 | oxidoreductase activity. acting on paired donors. with incorporation or reduction of molecular oxygen. NADH or NADPH as one donor. and incorporation of one atom of oxygen | 2.62E-03 | 0.025 | 20 | 748 | 7 |
| GO:0048565 | gut development | 2.66E-03 | 0.019 | 9 | 2800 | 8 |
| GO:0045596 | negative regulation of cell differentiation | 2.66E-03 | 0.043 | 67 | 991 | 18 |
| GO:0009395 | phospholipid catabolic process | 2.76E-03 | 0.019 | 7 | 1311 | 5 |
| GO:0048584 | positive regulation of response to stimulus | 2.82E-03 | 0.045 | 92 | 3140 | 53 |
| GO:0032640 | tumor necrosis factor production | 2.90E-03 | 0.022 | 16 | 1282 | 8 |
| GO:0002764 | immune response-regulating signal transduction | 2.95E-03 | 0.043 | 43 | 527 | 9 |
| GO:0001974 | blood vessel remodeling | 3.05E-03 | 0.008 | 6 | 1681 | 5 |
| GO:0050869 | negative regulation of B cell activation | 3.07E-03 | 0.009 | 7 | 3212 | 7 |
| GO:0032760 | positive regulation of tumor necrosis factor production | 3.15E-03 | 0.024 | 13 | 1282 | 7 |
| GO:0007406 | negative regulation of neuroblast proliferation | 3.15E-03 | 0.026 | 12 | 3669 | 11 |
| GO:0045885 | positive regulation of survival gene product expression | 3.20E-03 | 0.018 | 8 | 2528 | 7 |
| GO:0009897 | external side of plasma membrane | 3.23E-03 | 0.049 | 72 | 1169 | 21 |
| GO:0045577 | regulation of B cell differentiation | 3.26E-03 | 0.024 | 19 | 2954 | 14 |
| GO:0016627 | oxidoreductase activity. acting on the CH-CH group of donors | 3.52E-03 | 0.043 | 44 | 528 | 9 |
| GO:0008060 | ARF GTPase activator activity | 3.59E-03 | 0.037 | 19 | 1936 | 11 |
| GO:0032312 | regulation of ARF GTPase activity | 3.59E-03 | 0.038 | 19 | 1936 | 11 |
| GO:0007405 | neuroblast proliferation | 3.66E-03 | 0.034 | 15 | 3669 | 13 |
| GO:0001889 | liver development | 3.78E-03 | 0.031 | 14 | 358 | 4 |
| GO:0034329 | cell junction assembly | 3.88E-03 | 0.025 | 11 | 472 | 4 |
| GO:0002347 | response to tumor cell | 3.89E-03 | 0.021 | 8 | 3669 | 8 |
| GO:0042035 | regulation of cytokine biosynthetic process | 3.96E-03 | 0.035 | 34 | 1475 | 14 |
| GO:0033180 | proton-transporting V-type ATPase. V1 domain | 4.00E-03 | 0.029 | 8 | 2615 | 7 |
| GO:0045088 | regulation of innate immune response | 4.14E-03 | 0.041 | 15 | 1129 | 7 |
| GO:0002833 | positive regulation of response to biotic stimulus | 4.24E-03 | 0.018 | 6 | 2954 | 6 |
| GO:0002836 | positive regulation of response to tumor cell | 4.24E-03 | 0.02 | 6 | 2954 | 6 |
| GO:0002837 | regulation of immune response to tumor cell | 4.24E-03 | 0.022 | 6 | 2954 | 6 |
| GO:0002834 | regulation of response to tumor cell | 4.24E-03 | 0.022 | 6 | 2954 | 6 |
| GO:0002418 | immune response to tumor cell | 4.24E-03 | 0.025 | 6 | 2954 | 6 |
| GO:0002839 | positive regulation of immune response to tumor cell | 4.24E-03 | 0.025 | 6 | 2954 | 6 |
| GO:0032623 | interleukin-2 production | 4.30E-03 | 0.037 | 12 | 3082 | 10 |
| GO:0008329 | pattern recognition receptor activity | 4.35E-03 | 0.03 | 9 | 2208 | 7 |
| GO:0006014 | D-ribose metabolic process | 4.36E-03 | 0.022 | 6 | 2967 | 6 |
| GO:0022409 | positive regulation of cell-cell adhesion | 4.36E-03 | 0.036 | 11 | 3555 | 10 |
| GO:0046626 | regulation of insulin receptor signaling pathway | 4.62E-03 | 0.025 | 6 | 474 | 3 |
| GO:0046627 | negative regulation of insulin receptor signaling pathway | 4.62E-03 | 0.028 | 6 | 474 | 3 |
| GO:0042100 | B cell proliferation | 4.63E-03 | 0.038 | 20 | 3212 | 15 |
| GO:0043020 | NADPH oxidase complex | 4.64E-03 | 0.026 | 6 | 1034 | 4 |
| GO:0040018 | positive regulation of multicellular organism growth | 4.66E-03 | 0.026 | 7 | 1468 | 5 |
| GO:0048566 | embryonic gut development | 5.00E-03 | 0.025 | 6 | 1865 | 5 |
| GO:0006997 | nucleus organization | 5.17E-03 | 0.04 | 16 | 2954 | 12 |
| GO:0043648 | dicarboxylic acid metabolic process | 5.26E-03 | 0.042 | 17 | 2725 | 12 |
| GO:0002863 | positive regulation of inflammatory response to antigenic stimulus | 6.20E-03 | 0.047 | 11 | 2954 | 9 |
| GO:0050798 | activated T cell proliferation | 6.20E-03 | 0.047 | 11 | 2954 | 9 |
| GO:0042092 | T-helper 2 type immune response | 6.24E-03 | 0.045 | 13 | 222 | 3 |
| GO:0032606 | type I interferon production | 6.80E-03 | 0.034 | 7 | 940 | 4 |
| GO:0045351 | type I interferon biosynthetic process | 6.80E-03 | 0.037 | 7 | 940 | 4 |
| GO:0030865 | cortical cytoskeleton organization | 6.91E-03 | 0.036 | 10 | 1015 | 5 |
| GO:0030018 | Z disc | 7.18E-03 | 0.048 | 12 | 826 | 5 |
| GO:0033081 | regulation of T cell differentiation in the thymus | 7.34E-03 | 0.039 | 6 | 1169 | 4 |
| GO:0006573 | valine metabolic process | 8.22E-03 | 0.039 | 7 | 2528 | 6 |
| GO:0032615 | interleukin-12 production | 8.27E-03 | 0.036 | 6 | 2075 | 5 |
| GO:0055088 | lipid homeostasis | 8.85E-03 | 0.043 | 8 | 2954 | 7 |
| GO:0055092 | sterol homeostasis | 8.85E-03 | 0.046 | 8 | 2954 | 7 |
| GO:0042632 | cholesterol homeostasis | 8.85E-03 | 0.047 | 8 | 2954 | 7 |
| GO:0016500 | protein-hormone receptor activity | 9.12E-03 | 0.042 | 6 | 2119 | 5 |
| GO:0042535 | positive regulation of tumor necrosis factor biosynthetic process | 1.03E-02 | 0.039 | 6 | 1282 | 4 |
| GO:0042533 | tumor necrosis factor biosynthetic process | 1.03E-02 | 0.045 | 6 | 1282 | 4 |
| GO:0002762 | negative regulation of myeloid leukocyte differentiation | 1.03E-02 | 0.047 | 6 | 1282 | 4 |
