# Supplementary material for: Partial Sleep Restriction Activates Immune Response-Related Gene Expression Pathways: Experimental and Epidemiological Studies in Humans
Source: PLoS One. 2013 Oct 23;8(10):e77184. doi: 10.1371/journal.pone.0077184 (PMC3806729; doi:10.1371/journal.pone.0077184)
Supplement: Table S5 — Biological pathways down-regulated after sleep restriction. Gene Ontology pathways (biological processes) that were significantly enriched (P<0.05 after permutation) among the transcripts down-regulated after sleep restriction. Total no. of genes in pathway represents the number of genes that are annotated to the pathway. Top no. of genes in pathway represents the number of genes that were found changed in the study setting and contributed to the significance of the pathway. (DOCX) [file pone.0077184.s005.docx]

**Table S5.** Gene Ontology pathways (biological processes) that were significantly enriched (*P*<0.05 after permutation) among the transcripts down-regulated after sleep restriction. *Total no. of genes in pathway* represents the number of genes that are annotated to the pathway. *Top no. of genes in pathway* represents the number of genes that were found in the study setting and contributed to the significance of the pathway.

| **Gene Ontology ID** | **Pathway** | **Opt. *P* value** | **Permuted *P* value** | **Total no. of genes in pathway** | **Gene rank** | **Top no. of genes in pathway** |
| --- | --- | --- | --- | --- | --- | --- |
| GO:0032365 | intracellular lipid transport | 1.71E-05 | 0.001 | 9 | 124 | 4 |
| GO:0015833 | peptide transport | 1.48E-04 | 0.003 | 20 | 88 | 4 |
| GO:0015918 | sterol transport | 1.79E-04 | 0.001 | 8 | 95 | 3 |
| GO:0055092 | sterol homeostasis | 1.79E-04 | 0.001 | 8 | 95 | 3 |
| GO:0030301 | cholesterol transport | 1.79E-04 | 0.001 | 8 | 95 | 3 |
| GO:0042632 | cholesterol homeostasis | 1.79E-04 | 0.001 | 8 | 95 | 3 |
| GO:0045736 | negative regulation of cyclin-dependent protein kinase activity | 2.91E-04 | 0.003 | 9 | 495 | 5 |
| GO:0006869 | lipid transport | 3.71E-04 | 0.006 | 49 | 124 | 6 |
| GO:0055088 | lipid homeostasis | 3.75E-04 | 0.002 | 10 | 95 | 3 |
| GO:0005548 | phospholipid transporter activity | 3.91E-04 | 0.006 | 14 | 67 | 3 |
| GO:0005319 | lipid transporter activity | 4.91E-04 | 0.004 | 25 | 95 | 4 |
| GO:0015909 | long-chain fatty acid transport | 5.82E-04 | 0.005 | 9 | 124 | 3 |
| GO:0008299 | isoprenoid biosynthetic process | 7.30E-04 | 0.004 | 7 | 2235 | 7 |
| GO:0032393 | MHC class I receptor activity | 7.52E-04 | 0.005 | 7 | 1418 | 6 |
| GO:0006635 | fatty acid beta-oxidation | 8.20E-04 | 0.01 | 10 | 124 | 3 |
| GO:0004715 | non-membrane spanning protein tyrosine kinase activity | 8.23E-04 | 0.011 | 16 | 1756 | 11 |
| GO:0050680 | negative regulation of epithelial cell proliferation | 1.04E-03 | 0.012 | 15 | 87 | 3 |
| GO:0015908 | fatty acid transport | 1.11E-03 | 0.007 | 11 | 124 | 3 |
| GO:0009062 | fatty acid catabolic process | 1.11E-03 | 0.008 | 11 | 124 | 3 |
| GO:0004860 | protein kinase inhibitor activity | 1.17E-03 | 0.018 | 19 | 279 | 5 |
| GO:0016538 | cyclin-dependent protein kinase regulator activity | 1.26E-03 | 0.009 | 11 | 529 | 5 |
| GO:0048286 | alveolus development | 1.28E-03 | 0.01 | 7 | 514 | 4 |
| GO:0030509 | BMP signaling pathway | 1.29E-03 | 0.011 | 13 | 109 | 3 |
| GO:0007623 | circadian rhythm | 1.45E-03 | 0.015 | 14 | 2720 | 12 |
| GO:0046395 | carboxylic acid catabolic process | 1.46E-03 | 0.006 | 12 | 124 | 3 |
| GO:0016054 | organic acid catabolic process | 1.46E-03 | 0.007 | 12 | 124 | 3 |
| GO:0019210 | kinase inhibitor activity | 1.51E-03 | 0.018 | 20 | 279 | 5 |
| GO:0000122 | negative regulation of transcription from RNA polymerase II promoter | 1.53E-03 | 0.043 | 92 | 186 | 9 |
| GO:0045047 | protein targeting to ER | 1.58E-03 | 0.011 | 13 | 977 | 7 |
| GO:0051180 | vitamin transport | 1.75E-03 | 0.014 | 7 | 241 | 3 |
| GO:0016197 | endosome transport | 1.89E-03 | 0.028 | 34 | 99 | 4 |
| GO:0006790 | sulfur metabolic process | 2.01E-03 | 0.019 | 34 | 47 | 3 |
| GO:0050678 | regulation of epithelial cell proliferation | 2.13E-03 | 0.017 | 19 | 87 | 3 |
| GO:0050673 | epithelial cell proliferation | 2.13E-03 | 0.017 | 19 | 87 | 3 |
| GO:0042476 | odontogenesis | 2.31E-03 | 0.015 | 9 | 1707 | 7 |
| GO:0046486 | glycerolipid metabolic process | 2.39E-03 | 0.01 | 7 | 1086 | 5 |
| GO:0051181 | cofactor transport | 2.73E-03 | 0.019 | 8 | 241 | 3 |
| GO:0004896 | hematopoietin/interferon-class (D200-domain) cytokine receptor activity | 2.80E-03 | 0.028 | 23 | 1936 | 14 |
| GO:0048511 | rhythmic process | 2.97E-03 | 0.035 | 23 | 2720 | 17 |
| GO:0045639 | positive regulation of myeloid cell differentiation | 3.33E-03 | 0.04 | 16 | 861 | 7 |
| GO:0004861 | cyclin-dependent protein kinase inhibitor activity | 3.52E-03 | 0.024 | 9 | 495 | 4 |
| GO:0050663 | cytokine secretion | 4.04E-03 | 0.035 | 12 | 177 | 3 |
| GO:0034703 | cation channel complex | 4.07E-03 | 0.032 | 21 | 1983 | 13 |
| GO:0030324 | lung development | 4.23E-03 | 0.048 | 20 | 514 | 6 |
| GO:0004908 | interleukin-1 receptor activity | 4.46E-03 | 0.024 | 7 | 1936 | 6 |
| GO:0019966 | interleukin-1 binding | 4.46E-03 | 0.028 | 7 | 1936 | 6 |
| GO:0019965 | interleukin binding | 4.59E-03 | 0.036 | 15 | 1936 | 10 |
| GO:0004907 | interleukin receptor activity | 4.59E-03 | 0.039 | 15 | 1936 | 10 |
| GO:0001709 | cell fate determination | 4.70E-03 | 0.04 | 12 | 944 | 6 |
| GO:0007034 | vacuolar transport | 4.82E-03 | 0.023 | 11 | 207 | 3 |
| GO:0006662 | glycerol ether metabolic process | 5.47E-03 | 0.032 | 8 | 1086 | 5 |
| GO:0050918 | positive chemotaxis | 5.49E-03 | 0.036 | 9 | 3516 | 9 |
| GO:0001763 | morphogenesis of a branching structure | 6.18E-03 | 0.045 | 13 | 1590 | 8 |
| GO:0006720 | isoprenoid metabolic process | 6.24E-03 | 0.037 | 12 | 683 | 5 |
| GO:0042446 | hormone biosynthetic process | 7.14E-03 | 0.033 | 9 | 2041 | 7 |
| GO:0005112 | Notch binding | 7.45E-03 | 0.031 | 7 | 400 | 3 |
| GO:0050927 | positive regulation of positive chemotaxis | 9.79E-03 | 0.047 | 8 | 3516 | 8 |
| GO:0060113 | inner ear receptor cell differentiation | 1.14E-02 | 0.038 | 8 | 400 | 3 |
| GO:0016601 | Rac protein signal transduction | 1.25E-02 | 0.049 | 7 | 2327 | 6 |
