# Supplementary material for: Partial Sleep Restriction Activates Immune Response-Related Gene Expression Pathways: Experimental and Epidemiological Studies in Humans
Source: PLoS One. 2013 Oct 23;8(10):e77184. doi: 10.1371/journal.pone.0077184 (PMC3806729; doi:10.1371/journal.pone.0077184)
Supplement: Table S6 — Toll-like receptor (TLR) coding genes up-regulated after sleep restriction. The expression levels of several transcripts coding for TLRs were up-regulated after experimental sleep restriction (SR) compared to baseline (BL) values with pointwise t test (P<0.05). (DOCX) [file pone.0077184.s006.docx]

**Table S6.** Several Toll-like receptor (TLR)-coding genes were up-regulated after sleep restriction.

The expression levels of several transcripts coding for TLRs were up-regulated after experimental sleep restriction (SR) compared to baseline values (BL) with *t* test (pairwise *P*<0.05).

|  |  | **SR to BL** | |
| --- | --- | --- | --- |
| **Affymetrix Probe** | **Gene symbol** | ***P* value** | **Fold change** |
| 204924_at | **TLR2** | 0.0033 | 1.28 |
| 221060_s_at | **TLR4** | 0.0218 | 1.91 |
| 1552798_a_at | **TLR4** | 0.0432 | 1.73 |
| 220146_at | **TLR7** | 0.0067 | 1.47 |
| 220832_at | **TLR8** | 0.0245 | 2.10 |
| 229560_at | **TLR8** | 0.0298 | 1.75 |
